# Development of biomass waste-based carbon quantum dots and their potential application as non-toxic bioimaging agents†

**DOI:** 10.1039/d3ra05840a

**Published:** 2023-09-25

**Authors:** Norhidayah Abu, Shanmugavel Chinnathambi, Mahima Kumar, Fatemeh Etezadi, Noremylia Mohd Bakhori, Zuhana Ahmad Zubir, Shahrul Nizam Md Salleh, Rafidah Hanim Shueb, Subramani Karthikeyan, Vaijayanthi Thangavel, Jaafar Abdullah, Ganesh N. Pandian

**Affiliations:** a Department of Medical Microbiology & Parasitology, School of Medical Sciences, Universiti Sains Malaysia, Health Campus Kubang Kerian 16150 Kelantan Malaysia nhidayah@sirim.my; b Advanced Materials Research Centre (AMREC), SIRIM Berhad Lot 34, Jalan Hi-Tech 2/3, Kulim, Hi-Tech Park 09000 Kulim Malaysia; c Institute for Integrated Cell-Material Sciences, Institute for Advanced Study, Kyoto University Kyoto Japan chinnathambi.shanmugavel.8s@kyoto-u.ac.jp namasivayam.ganeshpandian.5z@kyoto-u.ac.jp; d Centre for Healthcare Advancement, Innovation and Research, Vellore Institute of Technology Chennai 600 127 India; e Department of Chemistry, Faculty of Science, Universiti Putra Malaysia, UPM Serdang Serdang 43400 Selangor Malaysia jafar@upm.edu.my

## Abstract

Over recent years, carbon quantum dots (CQDs) have advanced significantly and gained substantial attention for their numerous benefits. These benefits include their simple preparation, cost-effectiveness, small size, biocompatibility, bright luminescence, and low cytotoxicity. As a result, they hold great potential for various fields, including bioimaging. A fascinating aspect of synthesizing CQDs is that it can be accomplished by using biomass waste as the precursor. Furthermore, the synthesis approach allows for control over the physicochemical characteristics. This paper unequivocally examines the production of CQDs from biomass waste and their indispensable application in bioimaging. The synthesis process involves a simple one-pot hydrothermal method that utilizes biomass waste as a carbon source, eliminating the need for expensive and toxic reagents. The resulting CQDs exhibit tunable fluorescence and excellent biocompatibility, making them suitable for bioimaging applications. The successful application of biomass-derived CQDs has been demonstrated through biological evaluation studies in various cell lines, including HeLa, Cardiomyocyte, and iPS, as well as in medaka fish eggs and larvae. Using biomass waste as a precursor for CQDs synthesis provides an environmentally friendly and sustainable alternative to traditional methods. The resulting CQDs have potential applications in various fields, including bioimaging.

## Abstract 2

This paper unequivocally examines the production of carbon quantum dots from biomass waste, such as palm kernel shells and oyster shells, and their application in bioimaging. We also prepared carbon quantum dots from citric acid as a comparison.

## Graphical abstract

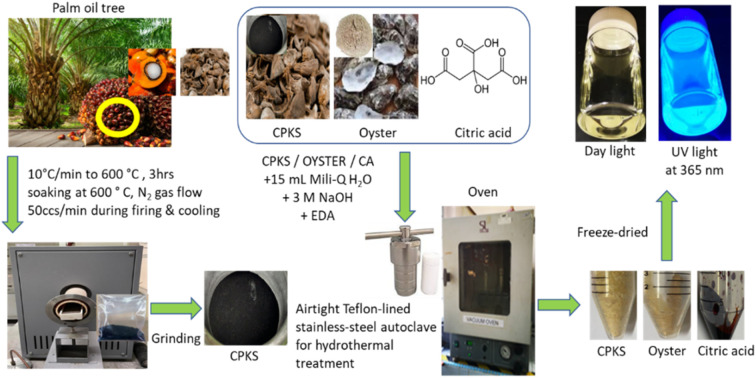

## 1. Introduction

Agricultural and food waste from farming and processing industries unequivocally represent significant pollutants in land and water systems. The consequences of these biomass wastes are severe and gravely impact the well-being of living beings. Every year, the global trade of crustaceans produces 6 to 8 million tonnes of discarded shells from valuable species such as shrimp, lobster, oyster, and crab.^1^ Seafood processing plants have been disposing of their residue in landfills, which could lead to environmental issues such as water and soil pollution. Furthermore, the disposal of this waste can attract pests such as flies and mosquitoes, spread harmful pathogens, promote bacterial growth, emit unpleasant odors, and create a visual nuisance for nearby residents.^2^ The oil palm industry in Malaysia, with 5.6 million hectares of plantations, is the second largest in the world and produces large amounts of waste every day.^3^ After the oil palm fruits are harvested and crushed to extract the oils, they will produce oil palm fibers, palm kernel shells, and empty fruit bunches as wastes.^4^ Malaysia's abundant natural resources have led to the exploration of using biomass waste as a foundation for creating innovative nanomaterials, promoting the “from waste to wealth” concept in the field of nanotechnology. One of the ingenious ways to utilize biomass waste from oil palm waste or seafood waste is to convert them as a carbon precursor in synthesizing carbon-based quantum dots.

Carbon-based quantum dots (CQDs) are novel metal-free quantum dots with sizes typically less than 10 nm with either an amorphous or crystalline structure.^5–8^ Such strong size dependencies facilitate fine-tuning the quantum dots' emission wavelength over almost the entire visible spectrum. Excitation-wavelength-dependent photoluminescence (PL), which implies that the emission wavelength and intensity can be controlled by changing the excitation wavelength, is an intriguing character that is often observed for CQDs. The unique properties such as excellent optical absorptivity, high hydrophilicity, photostable, cheap production, environment friendly, and non-toxic make them promising substitutes for conventional metal-based semiconductors and organic fluorophores.^9,10^ Researchers started to explore the applicability of CQDs in biochar absorbent,^11^ heavy metal detection.^12–18^ antibacterial agent,^19^ bioimaging.^16,20,21^ photocatalytic^22^ Numerous studies have successfully synthesized the CQDs from various sources such as crab-shell derived,^12^ lettuce waste,^11^ figs derived,^19^ durian shell waste,^13^ peach leaves waste,^20^ oil palm waste^10,16,17,23^ cashew gum,^24^ cellulose-based biowaste,^14^ semisolid waste of cow manure,^21,22^ green pepper,^15^ grapefruit peel,^25^ corn straw,^18^ fennel seeds^8^ chitosan, glucosamine, cellulose, glucose. The elements of C, H, O, and N are the primary elements found in CQDs, and the surface of CQDs can be functionalized with polar functional groups such as carboxyl, amine, and hydroxyl, which is beneficial for surface passivation, which will improve the solubility of the CQDs.^26–28^ CQDs can be doped with heteroatoms or grafted with other substances to boost the quantum yield or affinity to certain biomolecules.^29,30^ By grafting NH_2_ groups onto biological structures, amines such as ethylenediamine, poly(ethylenediamine), and trimethylamine can improve affinity to the structures.^31^ The addition of nitrogen atoms also can enhance the imagining resolution and resistance to photobleaching.^32–35^

Typically, there are many approaches used to prepare CQDs, either using synthetic or natural materials, such as laser ablation, arc discharge, electrochemical carbonization, hydrothermal treatment, and microwave pyrolysis techniques.^36,37^ Among all, the hydrothermal method is a relatively facile and commonly employed method to synthesize CQDs with the advantages of being inexpensive, non-toxicity, and sustainable.^38,39^ In general, hydrothermal treatment is a one-step process *via* a chemical reaction that requires the mixture of the solutions to be heated above the ambient temperature and pressure in an enclosed Teflon-lined stainless-steel chamber as the reaction will induce self-passivation.^40–42^ The optical and electronic properties of the CQDs can be adjusted by varying the concentration of precursor, type and concentration of dopant, reaction time, reaction temperature, pH of the reaction system, and the most critical parameter is the type of solvent used.^43–49^

Citric acid (CA) is a common and easily obtainable weak organic acid widely used as a carbon precursor for producing fluorescent CQDs. There is a wealth of scientific literature on this subject, making it a popular choice for researchers. A hydrothermal reaction was carried out by Meierhofer *et al.*^50^ to assess the effect of amine precursor on the optical properties of the CQDs in the reaction between CA nitrogen-doped with polyethyleneimine (PEI) and 2,3-aminopyridine (DAP). The presence of sp^2^-hybridized nitrogen in DAP-based CA results in a higher absorption band, causing a redshift. This leads to strong photoluminescence quantum yields (QY) of between 40% and 48% at neutral pH for both DAP-based CA and PEI-based CA. These findings suggest that amine precursor-based optical property adjustment is a viable possibility. By tuning the basic condition of both CQDs to pH 1 and pH 12, the QY showed a significant decrease of 9% to 21% due to the distinctive deprotonation sequences of the functional groups and the specific fluorophores. Hao *et al.*^51^ prepared CA-based CQDs in a three-neck round-bottom flask by heating up to 180 °C by varying the temperature ranging from 10 h, 20 h, 30 h, and 40 h with color changes from colorless to yellow, orange, and brown. They measured the effect of CQDs on scale-inhibition performance, and the CQDs at 30 h showed the best scale-inhibition efficiency. The pH of the CQDs was also altered from acidic to alkaline to form carboxyl groups on the surface of the particles. The increment of pH value agreed with the performance of the scale inhibition as the carboxyl groups are easier to dissolve and create anions in an alkaline environment and interact with scale ions. The effect of precursor concentration ranging from 0.1 M to 1.0 M on the size and optical properties of the CQDs was determined by Pimpang *et al.*^52^ The samples were prepared by stirring the solution of CA at a temperature of 250 °C. The morphology of the particles for all concentrations was quite similar, but the size of the particles decreased with increasing the CA concentration. The optical absorption also showed a similar trend as the blue shift can be seen with a decrease in average size and a similar trend can be seen as reported by Bak *et al.*^53^ Zheng *et al.*^54^ explored the effect of ethylene diamine derivatives as a passivation agent on the QY by thermal pyrolysis and investigated for the first time their application *in vivo* and *in vitro* bioimaging. They found that the QY for each dopant was 11.4%, 10.6%, and 9.8% for triethylenetetramine (TETA), tetraethylenepentamine (TEPA), and polyene polyamine (PEPA), respectively, once excited under visible light with low toxicity and good biocompatibility. Thus, thermal decomposition is the simplest route to obtain the fluorescent CQDs from citric acid as the citric acid will be dehydrated and reduced in the temperature range of 160–250 °C.

Herein, a facile hydrothermal method will be employed in preparing CQDs from the CA and biomass wastes, inclusive of palm kernel shell waste and oyster shell waste. Their behaviors on the optical and physicochemical characteristics are systematically studied. We reported the effect of ethylene diamine for surface passivation, varying temperature and reaction time, and pH of the solvent on their photoluminescence properties. The cytotoxicity, *in vitro*, and *in vivo* bioimaging of the synthesized CQDs were also explored using HeLa cells, human-induced pluripotent stem cell cultivation, and medaka fish culture, respectively. The resulting CQDs exhibit excellent optical properties and biocompatibility, demonstrating the feasibility of CQDs for bioimaging applications.

## 2. Materials and methods

### 2.1 Materials

The palm kernel shells and oysters used as the carbon precursor from biomass waste were collected from Perusahaan Minyak Sawit Bintang (Malaysia) and Fish Farm Kuala Muda (Malaysia), respectively. We purchased various chemicals and solutions for the experiment. These included Ultra-pure water (Milli-Q; resistivity of 18.2 2 mΩ cm^−1^) from Fisher Scientific in the United Kingdom, ethylenediamine (EDA) from Acros Organics in the USA, absolute ethanol (EtOH) from Merck in Germany, hydrochloric acid (HCl), citric acid monohydrate (C_6_H_8_O_7_·H_2_O), and sodium hydroxide (NaOH) from Merck in Germany. We also bought phosphate buffer from Sigma-Aldrich in Germany. For this research, we utilized only analytical-grade chemicals and reagents. Hoechst 33342 Solution (62249) and BioTracker 650 Red Nuclear Dye (SCT119) were purchased from Sigma-Aldrich, USA. MitoTracker Green (M7514) was purchased from Invitrogen, Japan.

### 2.2 Synthesis of CQDs using hydrothermal method

The palm kernel shell and oyster shell used in this research were washed thoroughly with distilled-deionized water, dried in an oven at 70 °C, and crushed into fine powder. The palm kernel shell needs to be carbonized in nitrogen gas at a flow rate of 50 ccs min^−1^ with a 10 °C min^−1^ rise for 3 h at a temperature of 600 °C. The oyster shell was ground using an industrial blender, and the powder was sieved into a size of less than 500 μm and kept in a closed container.

**Fig. 1:**
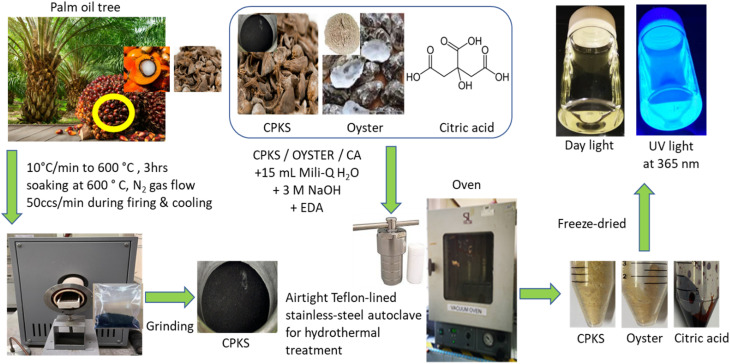
Carbon QDs synthesis steps from CPKS, oyster shells, and citric acid sources.

The amended synthesis steps were as follows: briefly, 0.3 g of CPKS was dissolved in a mixture of EDA (0.2 mL), 1.0 M NaOH (3 mL), deionized water (15 mL), and stirred for 30 minutes (QY: = 2.4%; Fig. S1†).^23^ The same method was applied in preparing the CQDs from oyster shells (QY = 1.4%; Fig. S2†). The CQDs derived from CA were prepared following Silvija *et al.*^55^ (QY = 22.1%: Fig. S3†) with slight modification by mixing CA (3.0 g) with EDA (1 mL) in deionized water (40 mL). The solutions were placed in an airtight Teflon-lined stainless-steel autoclave batch reactor with a 50 mL capacity. This was then subjected to hydrothermal treatment at 160 °C using a Protech Oven, Model FAC-50 from Malaysia. After finishing the process, the CQDs were filtered twice using a 0.22 μm filter syringe disc and a 20 mL syringe to eliminate micron-sized particles. The filtered CQDs solution was dialyzed using a dialysis tube (3.5 kDa) in a beaker wrapped with aluminum foil and left stirred overnight. The solutions were rotavapor for 1 h at 70 °C. The purified CQDs solution obtained was kept in a centrifuge tube (50 mL) and stored at −20 °C for freeze drying. The resulting purified CQD solutions were then stored at a temperature of 4 °C for future use (Fig. 1).

### 2.3 Characterization

The CQDs' PL emission spectra were measured at room temperature using the Fluorometer-Spark multimode microplate reader (Infinites F500, TECAN, Ltd, Mannedorf, Switzerland). In addition, the CQDs' PL excitation and emission spectra were measured by JASCO FP-8300 Fluorescence Spectrometer. Emission spectra were measured from 300 nm to 600 nm at room temperature on a JASCO FP-8300 spectrofluorometer. The emission spectra were recorded at a scan rate of 1000 nm min^−1^ with various excitation wavelengths. All samples were prepared in a total volume of 1.0 mL in Milli-Q water. Quantum yields were measured with a Hamamatsu Photonics Quantaurus-QY C11347-11 Absolute PL Quantum Yield Spectrometer and calculated with the integrated measurement software. At the same time, their absorbance was recorded by the UV-visible spectrophotometer (PerkinElmer Lambda 35, USA) within the 200 nm to 700 nm range. The functional groups on the CQDs were determined using a Fourier-transform infrared (FTIR) spectrophotometer (Thermo Scientific, USA), the particle size was captured by a high-resolution transmission electron microscopy (HR-TEM) (Philips, USA), and the surface chemistry of the CQDs was analyzed by X-ray photoelectron spectroscopy (XPS) (Omicron Nanotechnology, Germany).

### 2.4 HeLa cell culture

To conduct the *in vitro* studies, the HeLa cells were cultivated in a 75 cm^2^ flask under controlled conditions of 37 °C temperature, humidified air with 5% CO_2_, and provided with 10% fetal bovine serum, 5000 U per mL penicillin, and 50 mg mL^−1^ streptomycin. The DMEM medium is used for maintaining the HeLa cell lines. The sole supplier we relied on for tissue culture supplies was Fisher Scientific. For the assessment of cell cytotoxicity, the cells were cultivated on a 96-well plate with a density of 5000 cells per well. The cells were preincubated for 24 h and then treated with CQDs. The Cell Counting Kit-8 (Dojindo Laboratories, Osaka, Japan) was used to measure cell viability at 24 h and 48 h incubation periods. The absorbance at 450 nm was determined using a traditional microplate reader (MTP-880Lab; Corona, Hitachinaka, Japan). The mean ± standard deviation was utilized to present the results of the cell viability experiments, which were conducted in triplicate. The comparison of the percentage of cell cytotoxicity was made against the untreated control cells.

### 2.5 Human-induced pluripotent stem cells cultivation

The hiPSCs (253G1 cell line) were acquired from the RIKEN BioResource Center in Japan. These stem cells originate from human-induced pluripotent stem cells. They were cultured on tissue culture polystyrene, following previously described methods.^56^ The hiPSCs were cultured in a feeder-free environment using a TeSR-E8 culture medium from STEMCELL TECHNOLOGY (USA). The culture conditions involved maintaining the cells at 37 °C, with 5% CO_2_ and 95% humidity. The culture medium was refreshed daily, and cell passaging was performed when the cells reached 70% confluency. To determine cellular proliferation and viability, we analyzed the hiPSCs using the NucleoCounter NC-200 automated cell counter with Via 1-Cassette from ChemoMetec in Copenhagen, Denmark. We also visually inspected the adhesion and distribution of the hiPSCs with an IX71 inverted microscope from Olympus in the USA, equipped with a DP71 camera from Olympus. To calculate the cell-covered area, we used ImageJ software.

### 2.6 Differentiation of cardiomyocytes from hiPSCs

The differentiation of cardiomyocytes from hiPSCs (induced cardiomyocytes, iCMs) was conducted following the previously described method.^57^ When hiPSCs reached 90–100% confluence, they were induced into the mesoderm stage by introducing a medium consisting of B-27 supplemented-RPMI (which contains glucose but no insulin, Thermo Fisher Scientific, NY, USA) and 12 μM CHIR99021 (Wako, Japan). After precisely 24 h of CHIR99021 treatment, the CHIR99021 was removed. On the third day of differentiation, cardiac mesoderm induction commenced by adding a WNT inhibitor (2 μM XAV939, Wako). On the seventh day of differentiation, insulin (1 μg) was introduced, and the medium was changed every other day. It was anticipated that beating would occur sometime between the seventh and fourteenth day of differentiation.

### 2.7 Cell imaging

To visualize the CQDs in the cells, we used a confocal laser scanning fluorescence microscope (FluoView, FV10i, Olympus; Elyra 7 with Lattice SIM^2^, ZEISS) with 405 nm laser excitation. Also, we used a 488 nm laser for the mitochondria and a 640 nm/405 laser for the nucleus. We seeded HeLa, iPS, and cardiomyocytes in a 35 mm dish, and 24 h later, 100 μg mL^−1^ CQDs were added to a glass bottom dish as a final concentration. After a 24 h incubation period, the cells were washed in PBS and fixed for 10 minutes in 4% paraformaldehyde before confocal imaging. The cell nucleus was stained with Hoechst 33342 or BioTracker 650 Red Nuclear Dye.

### 2.8 Medaka fish culture

To conduct our *in vivo* experiments, we utilized embryos and larvae of medaka fish (Oryzias latipes). We obtained mature Oryzias latipes in Kyoto Prefecture from a local fish farm called Higo-pet. The fish must be housed in a glass aquarium filled with fresh water and kept at a strict temperature of 25 °C, with a 10 h dark cycle and a 14 h light cycle to perfectly replicate natural conditions. Any deviation from these conditions may hinder the reproduction process, and thus, artificial conditions must be created to ensure successful reproduction. As previously stated, we collected and monitored the spawned eggs in a Petri dish. We then seeded the eggs in triplicates onto a 96-well plate (one egg per well) and treated them at 4 days old. We continued to monitor them every day until day 28. Once the larvae hatched around day 17, we transferred and monitored them in a separate glass tank.

## 3. Results and discussion

### 3.1 Photophysical characterization

**Fig. 2:**
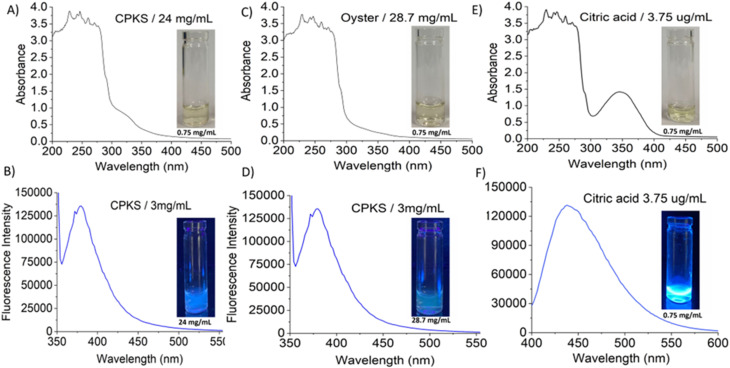
UV-visible and fluorescence spectrum of carbon quantum dots from three sources: CPKS (A and B), oyster shell (C and D), and citric acid (E and F). Inset images represent quantum dots under white light and UV light.

**Fig. 3:**
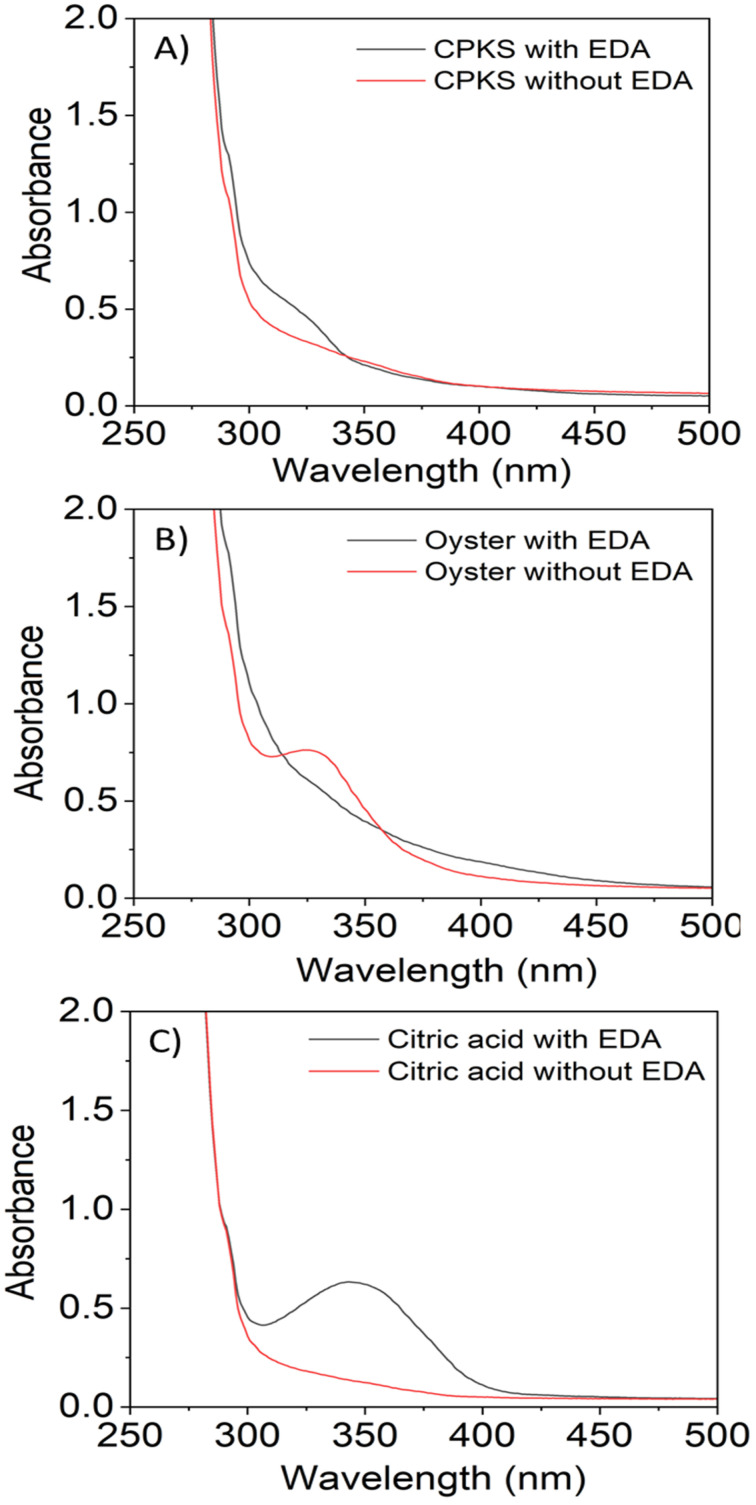
Effect of EDA *vs.* absorbance of carbon dots.

In this study, the CQDs have been successfully prepared from citric acid and biomass waste-based such as oyster shell and CPKS *via* hydrothermal treatment. The final products were freeze-dried and solubilized in water for further characterization. We studied the effect of temperature, pH, and precursor concentration on the synthesized CQDs' optical properties. The synthesized CQDs' UV-visible absorption and fluorescence emission spectra were used to understand their optical properties. Fig. 2A–F exhibited the absorption and fluorescence spectra of CQDs with the absorbance of the light between 300–400 nm and emission between 350–500 nm. The inset shows the photograph of CQDs under white light and UV light exposure. The UV visible absorption spectra showed not obvious with a minor shoulder at 280 nm for all CQDS, which was ascribed to the C=C bonds attributed to the π–π* electron transition.^58^ Similarly, for citric acid, the peak appeared at 345 nm. These peaks were attributed to the non-bonding electrons' n–π* transition in C–C, C–N, and C=O.^59,60^ The effect of the EDA as a precursor and freeze-drying process on the absorption of the CQDs is presented in Fig. S4† and 3. The absorbance spectra revealed there was no shifting in their absorption peak with the presence of EDA (Fig. 3). In contrast, the absorption spectrum of the CQDs becomes broad as the direct sublimation of the ice during freeze-drying process retains the particle dispersion, and the smaller spacing between the particles may lead to the emergence of new energy levels as a result of surface group interaction.^61^ The difference in the absorption intensity might be due to the concentration of the CQDs during the sample preparation for the UV-Vis spectrophotometry test.

**Fig. 4:**
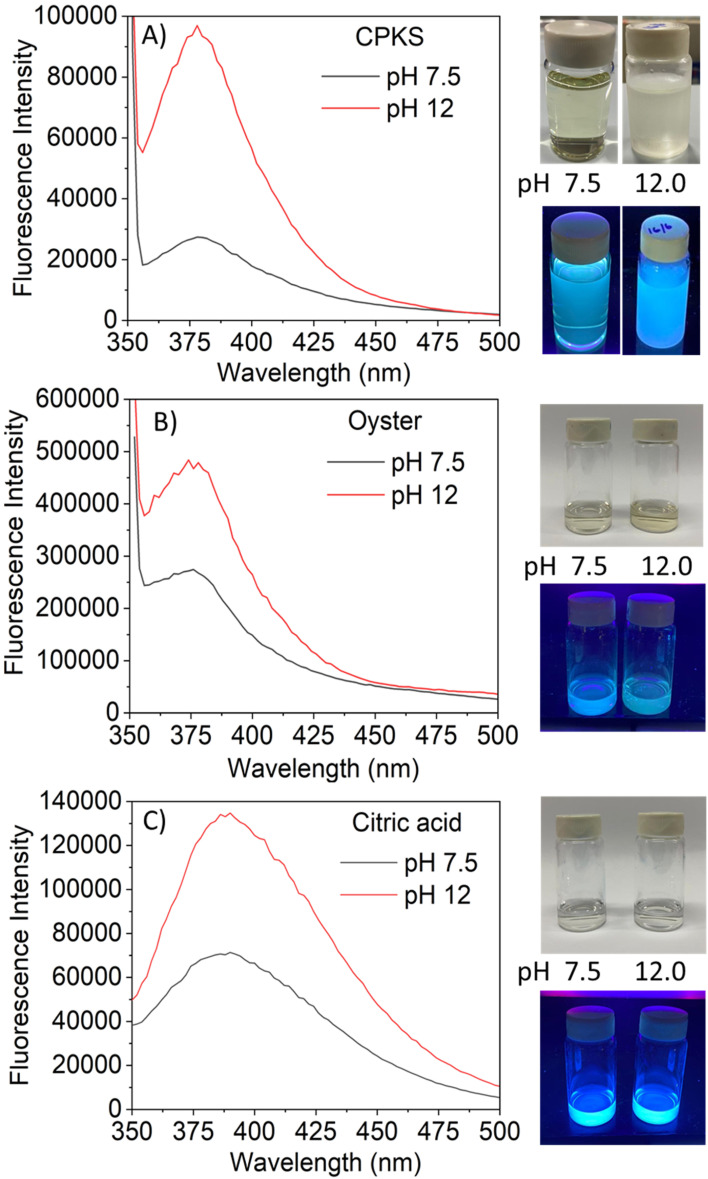
Effect of pH *vs.* fluorescence emission of carbon dots.

**Fig. 5:**
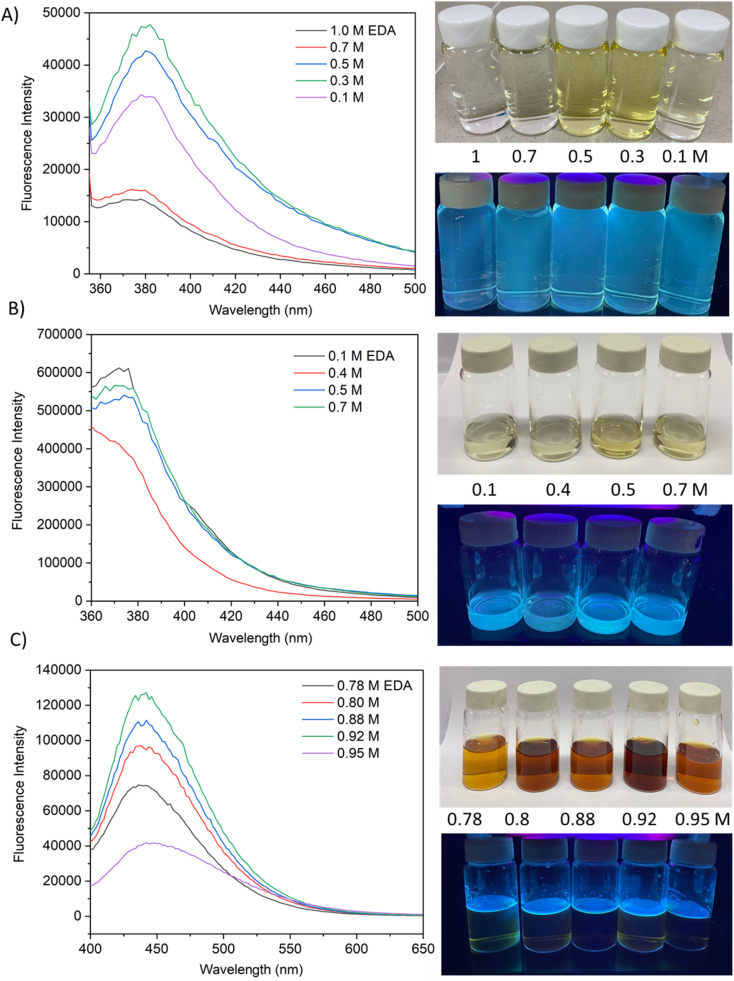
Effect of EDA concentration on fluorescence emission characteristics of carbon dots from CPKS, oyster shells, and citric acid sources.

**Fig. 6:**
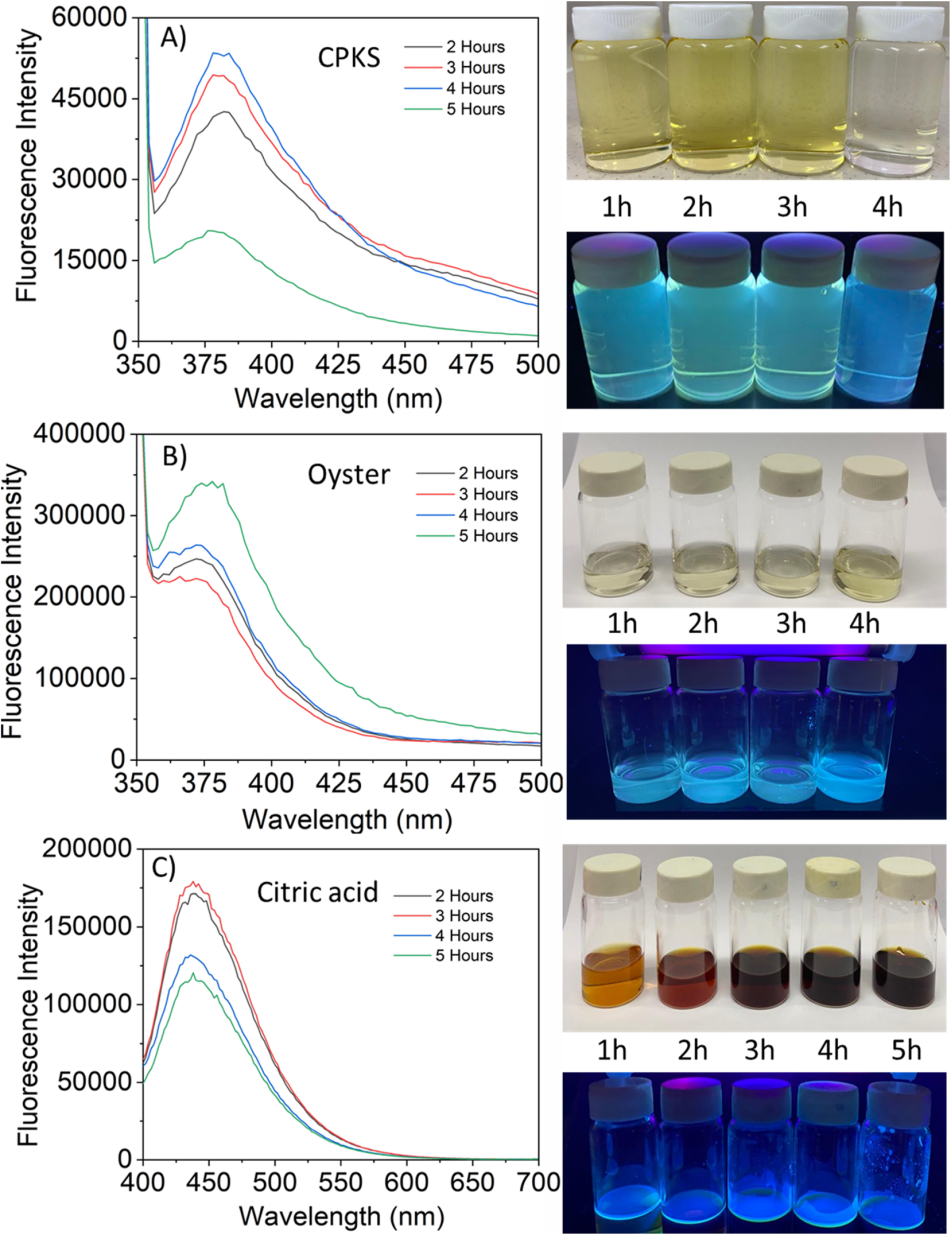
Effect of time *vs.* fluorescence of carbon dots.

The fluorescence emission spectrum of synthesized CQDs from CPKS (emission maximum at 380 nm), oyster shell (emission maximum at 380 nm), and citric acid (emission maximum at 438 nm) is shown in Fig. 2A–F, respectively. In the case of CPKS and oyster shell-based CQDs, we measured fluorescence emission spectra with 300, 325, and 350 nm excitation. We got very good intensity at 325 nm excitation compared with the other two excitations (Fig. S5 and S6†). In addition, we measured the fluorescence emission spectra of CAQDs with three excitation wavelengths (325, 350, and 375 nm). The emission wavelength and intensity aren't altered significantly (Fig. S7†). Compared with citric acid-based CQDs, the other two biomass waste-based CQDs showed narrow emission peaks. This narrow emissive material will be helpful for multicolor cell imaging without spectral overlap. For better fluorescence intensity, we prepared two samples with pH 7.5 and pH 12. Compared with pH 7, pH 12 shows high fluorescence intensity (Fig. 4). Unfortunately, we cannot use these samples for biological applications due to alkaline pH, but the pH 7.5 samples have enough fluorescence intensity for bioimaging. In addition, we optimized EDA precursor molar concentration to reach better fluorescence intensity. In the case of CPKS, an EDA precursor concentration of 0.3 M shows high fluorescence intensity, while oyster shell-based CQDs with a precursor concentration of 0.1 give high intensity. Finally, 0.92 M EDA gives better fluorescence for CQDs synthesized from CA (Fig. 5). We cannot determine the trend of the effect of the EDA precursor concentration on the fluorescence intensity; thus, the best EDA precursor concentration was selected based on the highest fluorescence intensity for optimization of reaction duration (Fig. 6). Based on the enhancement in the fluorescence intensity, the CPKS-based CQDs prepared in four hours reaction time showed better fluorescence agreed with reported by Monday *et al.*^23^ while the oyster shells and CA-based CQDs prepared in five hours and three hours reaction duration exhibited higher fluorescence intensity.

**Fig. 7:**
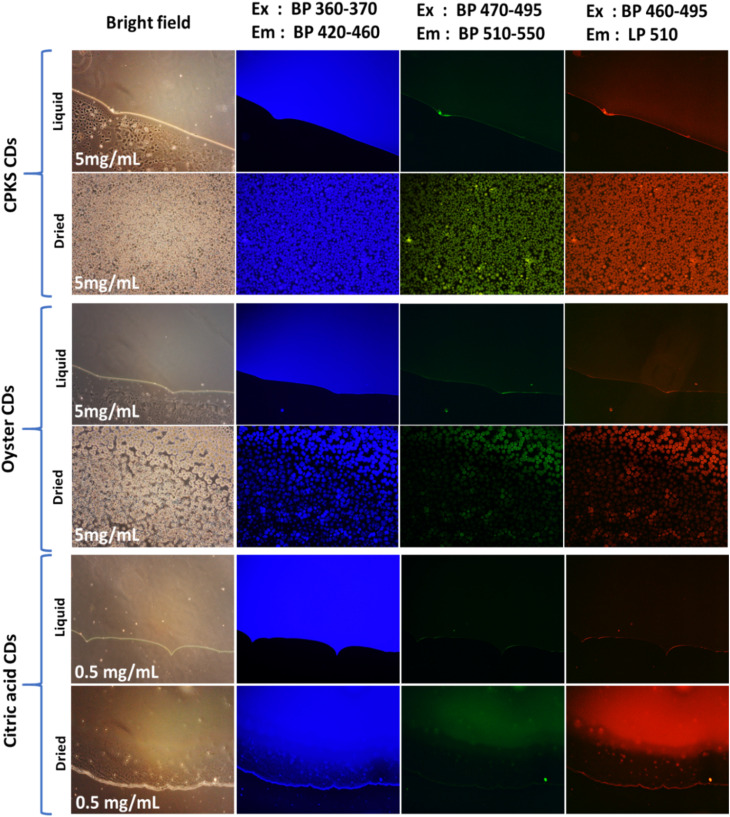
Fluorescence images of CPKS QDs, oyster QDs, and citric acid QDs excited with three different excitations and emission bandpass. The upper panel represents QDs in liquid form on the glass and covered by a coverslip. The lower panel represents QDs in dried condition.

The fluorescence images of CPKS-based CQDs dropped on the surface of the glass and covered with coverslip Fig. 7 (top panel), the lower panel shows CPKS-based CQDs dropped and dried at room temperature before observation. In the liquid stage, all the CQDs dispersed very well in water and dried condition, able to see CQDs accumulation. We used three different (blue, green, and red) excitation filters for fluorescence emission observation. Compared with green and red channels, blue channels give excellent light intensity. These results match the fluorescence emission spectra of CPKS-based CQDs. Similarly, we observed CQDs synthesized from oyster shells and CA and presented in Fig. 7 (middle and bottom panel).

### 3.2 High-resolution transmission electron microscopy (HR-TEM)

**Fig. 8:**
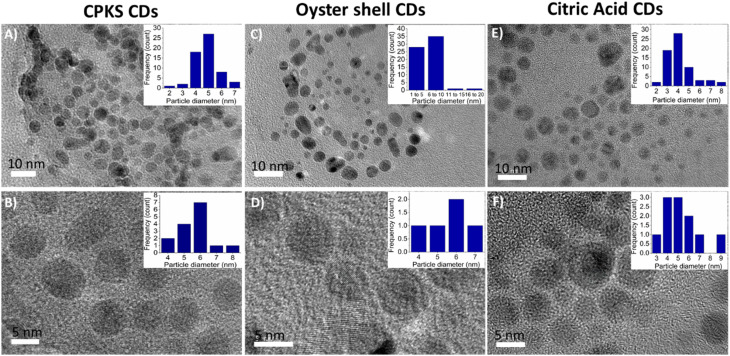
TEM images of carbon quantum dots synthesized from CPKS (A and B), oyster (C and D), and citric acid (E and F). Inset images represent the quantum dot's average sizes.

The HR-TEM was carried out to determine the morphological and particle size of three different CQDs presented in Fig. 8A–F, which were drop-cast from the water dispersion on a carbon-coated copper grid. Fig. 8(A, B), (C, D), and (E, F) represent CQDs derived from CPKS, oyster shells, and CA, respectively. It can be seen from the images that these CQDs have a roughly spherical form morphology and are evenly distributed without any aggregation. For the CQDs, the average size of the particles is 4.5 nm for CPKS, 6.0 nm for Oyster, and 5.0 nm for CA. High-resolution TEM (HR-TEM) images illustrate that most CQDs have well-resolved lattice structures with a *d* spacing value of 0.21 nm for CPKS, 0.23 nm for Oyster, and 0.26 nm for CA, in agreement with the (100) plane lattice of graphitic carbon.^62^

### 3.3 XPS analysis

XPS is a surface-sensitive technique investigating the binding energy and the chemical composition of the synthesized CQDs. After deconvoluting the XPS spectra, it was evident that the CQDs primarily consisted of oxygen, carbon, and nitrogen, as indicated by the three prominent peaks. In the XPS wide spectrum, three strong peaks were observed for CPKS (Fig. S8†), oyster shell (Fig. S9†), and CA (Fig. S10†) of CQDs. These peaks at 285, 399, and 531 eV were attributed to C 1s, N 1s, and O 1s, respectively. The relative atomic percentages of these peaks are summarized in Table S1.† It was reported that nitrogen bonded to carbon can improve the emission of nanoparticles. The deconvoluted peaks of C 1s represent π-bonded attributing to the sp^2^ and sp^3^-bonded carbon atom from C–C/C=C, C–N/C–O, C=O groups. The peaks of N 1s represent pyrrolic N and amine groups, and the peaks of O 1s are assigned to the binding energies of C=O, C–O, and the peak of 535 eV indicates the oxygen atom in their moieties like H_2_O and OH as the binding energy of water was sensitive to total surface coverage.^63^ The main component of N in the as-prepared CQDs is the pyrrolic N, formed through the dehydrolysis reaction between carboxyl and amine groups. According to previous reports, the pyrrolic N is believed to increase the electronic cloud density of CQD surfaces, ultimately enhancing luminescence efficiency.^64–66^

**Fig. 9:**
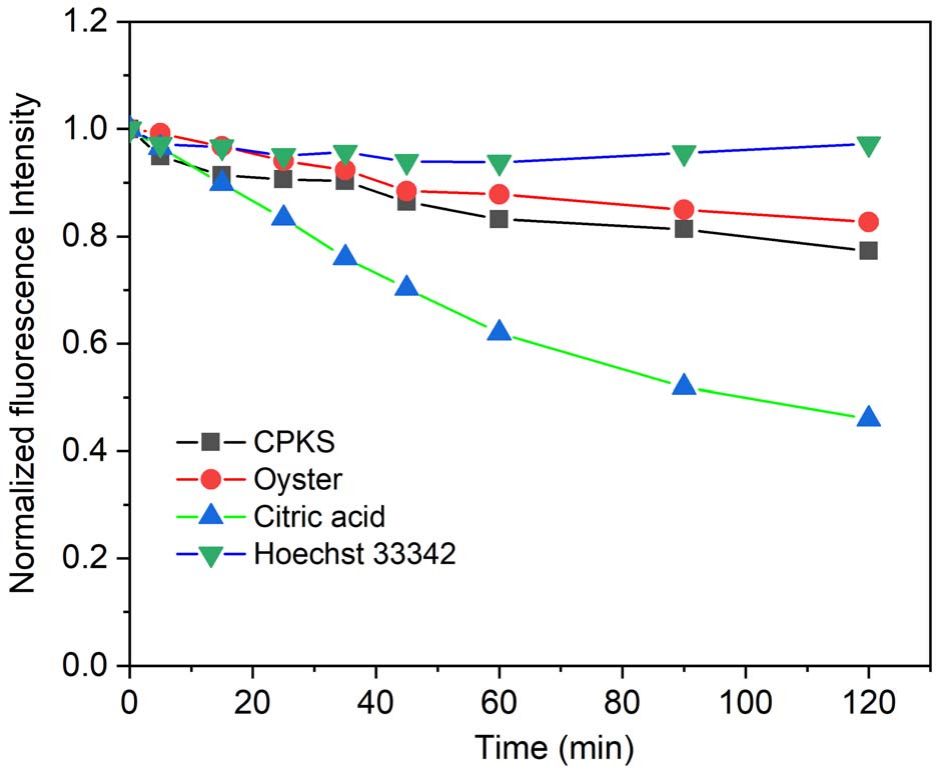
Quantum dot stability under 365 nm UV light exposure.

The CQDs produced had a multitude of O- and N-related functional groups responsible for their remarkable water solubility and could be strongly correlated with their fluorescence emission. The negative values for the as-prepared oyster shell, CPKS, and CA-based CQDs were further explained using the zeta potential depicted in Fig. S11.† They were found to be −56.1 ± 4.1 mV, −46.6 ± 2.6 mV, and −13.2 ± 0.8 mV, respectively. These values indicate a negative surface charge due to the presence of hydroxyl and carboxyl functional groups on the CQDs. The oyster shell and CPKS CQDs displayed significant negative zeta potential compared to CA-based CQDs due to numerous functional groups that contained oxygen by biomass waste. The quantum stability study showed that CQDs from oyster shells and CPKS were highly stable, as evidenced by a zeta potential greater than −30 mV even under UV light exposure (Fig. 9).

### 3.4 Photostability test under UV light

To check the photostability of the synthesized CQDs, we used 365 nm UV light irradiation concerning time. We used Hoechst 33342 nucleus staining dye for reference, which emits light in the blue region. In Fig. 9, Hoechst does not lose any fluorescence intensity after 120 minutes of continuous irradiation. Similarly, CPKS and Oysters also do not lose significant fluorescence intensity. In the case of CA QDs, the fluorescence intensity loss reaches up to 50%. Compared with CA CDs, the CPKS and oyster shell-based CQDs have more UV light stability. It also shows almost equal photostability with the commercial dye Hoechst 33342.

### 3.5 Fourier-transform infra-red (FTIR) spectroscopy

**Fig. 10:**
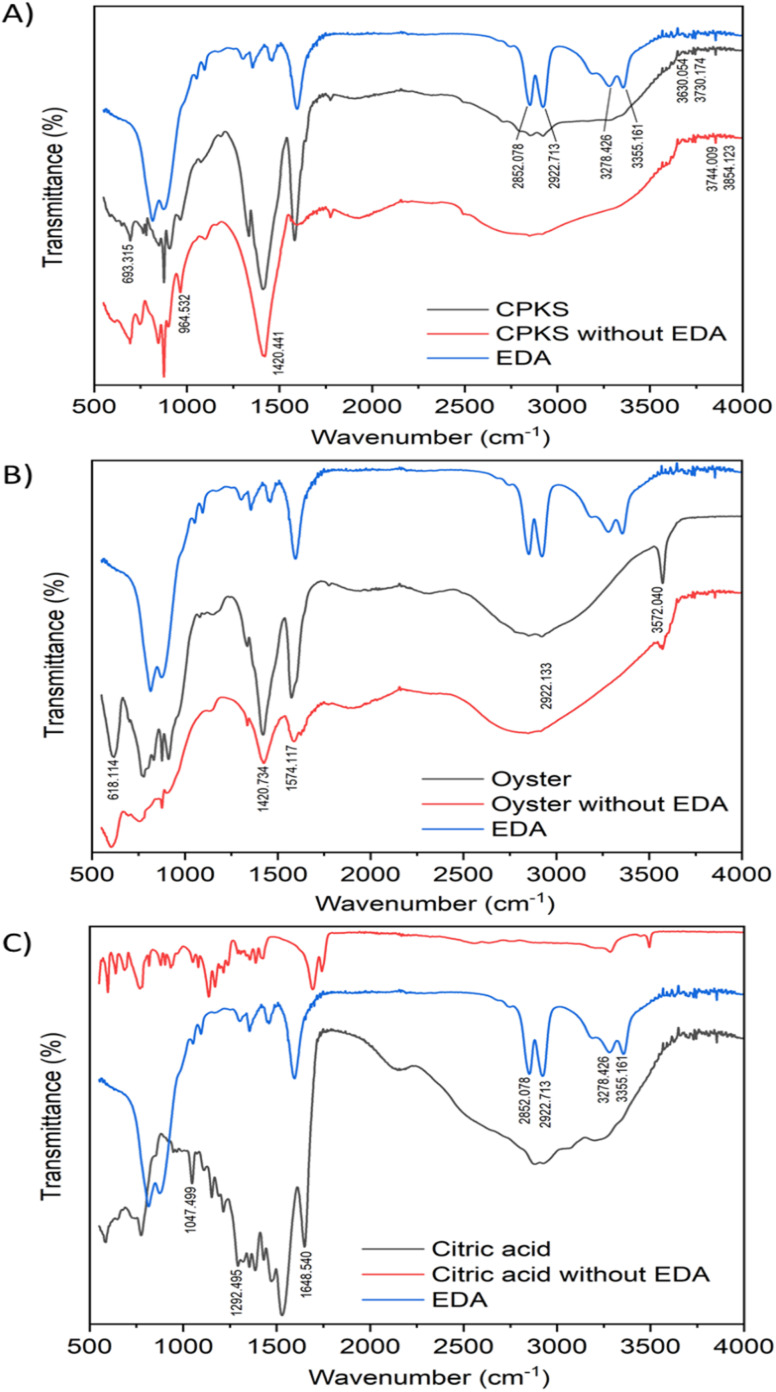
FTIR characterization of carbon quantum dots from three sources: CPKS (A), oyster shell (B), and citric acid (C).

The FTIR spectra of EDA, CQDs-EDA, and CQDs without EDA were shown in Fig. 10 to identify the different surface functional groups present on the CDs. The FTIR peaks in the range of 3000–3500 cm^−1^ correspond to the hydrophilic group O–H and N–H of primary aliphatic amines,^67^ hence explaining the excellent water solubility.^17^ The absorption bands at 2850–3000 cm^−1^ represent the C–H stretching vibration (sp^3^) assigned to CH_2_ groups, and the peak at 1600 cm^−1^ is related to the presence of the –NH_2_ scissoring vibration of a primary amine. The OH group was identified as the cause of the medium peak at 1420 cm^−1^, while the N–C bonds were responsible for the peak at 1350 cm^−1^. Additionally, the presence of peaks in the fingerprint region between 700–900 cm^−1^ may be attributed to the stretching vibrations of C–OH and the out-of-plane bending modes of C–H groups.^68^ Both the hydroxyl-functional group (–OH stretching) and amino-functional group (N–H stretching) were present in all CQDs, indicating the existence of amine in the EDA and their ability to dissolve in water. The amine bands are missing in the FTIR spectra of CQDs without EDA.

### 3.6 Cell viability and cellular uptake

**Fig. 11:**
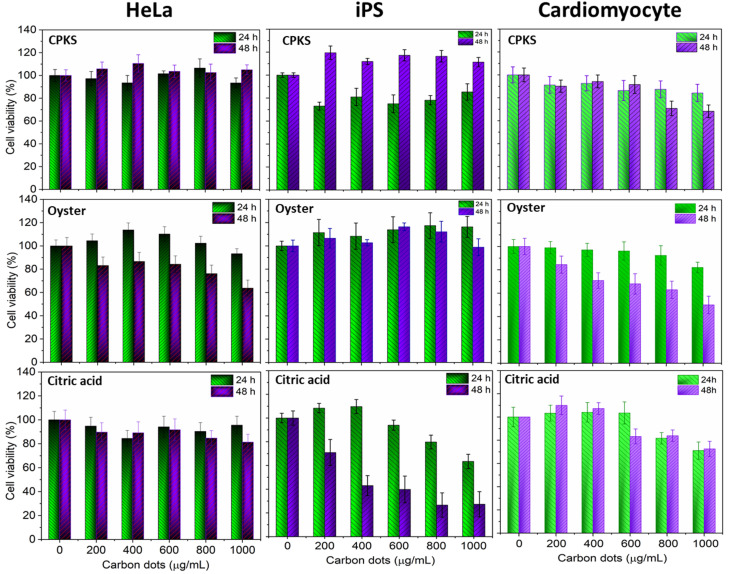
Cell viability assay using CPKS, oyster, and citric acid-based CQDs with HeLa, iPS, and cardiomyocyte cells. Cell viability was represented as mean ± SE (*n* = 3).

To verify the biocompatibility of CQDs in the *in vitro* models, we performed cellular viability and *in vitro* imaging studies using HeLa, iPS, and cardiomyocyte cells as representative cell lines. As shown in Fig. 11, HeLa cells were almost 100% viable after 24 h incubation, as no cytotoxicity was observed even with 1 mg mL^−1^ of CQDs from CPKA, oyster shell, and CA. Interestingly, in the case of CPKA, no toxicity is observed even after 48 h of incubation. In the case of CA-based CQDs, no cytotoxicity was observed after 24 h, but 48 h later, 15% toxicity was observed with higher concentrations (1 mg mL^−1^). Cell-viability studies indicate that functionalized CQDs are far less risky than heavy-metal quantum dots. Hence, they are the ideal choice for diagnostic studies that involve live cells, owing to their exceptional biocompatibility.

**Fig. 12:**
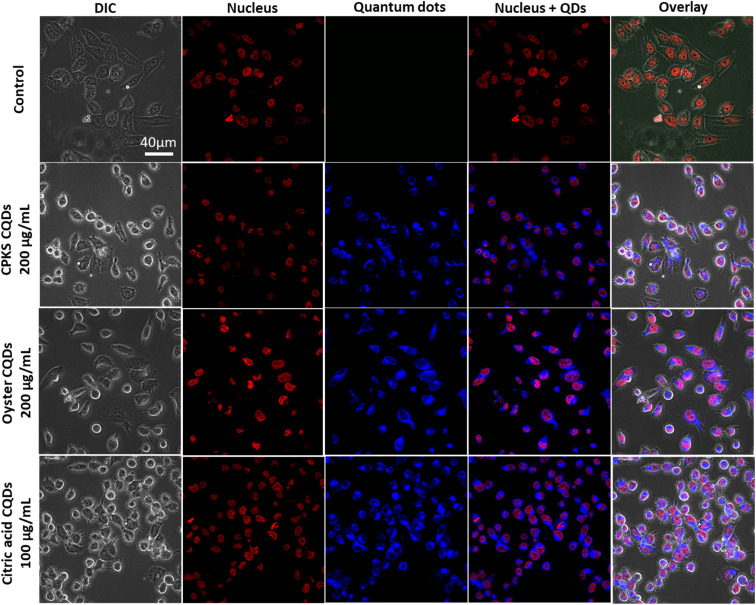
*In vitro* imaging studies using CPKS, oyster, and citric acid-based CQDs with HeLa cells.

**Fig. 13:**
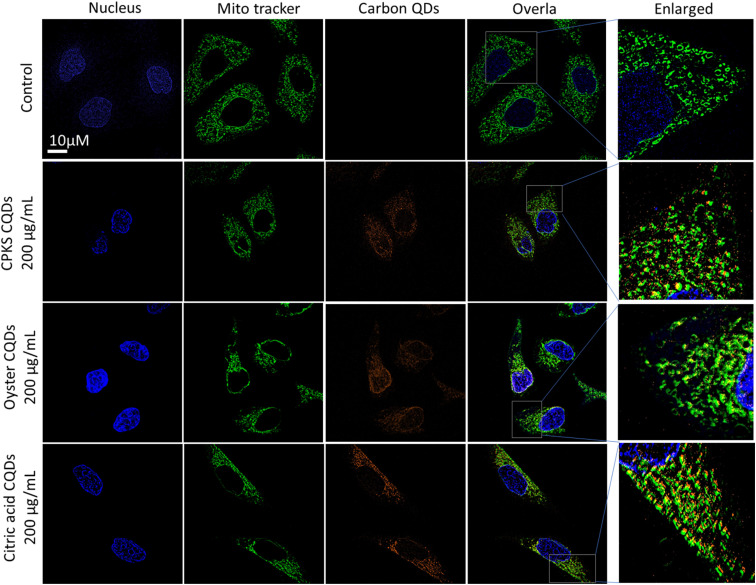
Mitochondrial localization using CPKS, oyster, and citric acid-based CQDs with HeLa cells.

Fig. 12 shows the laser scanning confocal images of the HeLa cells after CQDs incubation. The red represents the cell nucleus, and the blue shows CQDs. We used 200 μg mL^−1^ of CPKS and oyster shell-based CQDs for HeLa cell imaging. However, 100 μg mL^−1^ is used for citric acid-derived CQDs. Overlay images show that most CQDs are localized around the cell nucleus. To confirm the localization organelle of the CQDs, we used mito tracker dye, and both the CQDs and mito tracker merged very well (Fig. 13 and S12†). These results suggest that nitrogen-dopped CQDs can enter the mitochondria.

**Fig. 14:**
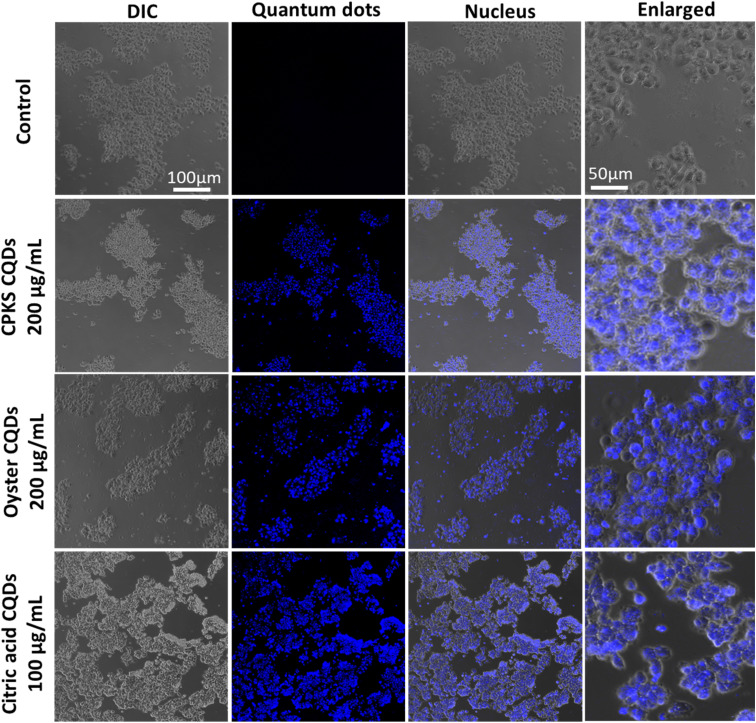
*In vitro* imaging studies using CPKS, oyster, and citric acid-based CQDs with iPS cells.

In the case of iPS cells (Fig. 14), CPKS-based CQDs do not show little cytotoxicity, even 200 μg mL^−1^ for 24 h. But, we cannot see any toxicity after 48 h. Oyster shell-based CQDs offer no cytotoxicity up to 1000 μg mL^−1^ in 24 and 48 h. No toxicity was observed for citric acid CQDs up to 600 μg mL^−1^ until 24 h. But 1000 μg mL^−1^ shows 35% of cell toxicity was observed. After 48 h, iPS cell shows more than 60% toxicity from 400 to 1000 μg mL^−1^. Fig. 14 shows the cellular uptake of CQDs by iPS cells. The iPS cells show very good cellular uptake throughout the cells. We used 200 μg mL^−1^ for CPKS and oyster shell CQDs, but we used only 100 μg mL^−1^ for CA-based CQDs due to high-level fluorescence capacity and low toxicity. We observed after 24 h of incubation with CQDs, even though we cannot see cell morphology changes. These results convinced that cell viability studies with <200 μg mL^−1^ of CQDs were nontoxic to iPS cells.

**Fig. 15:**
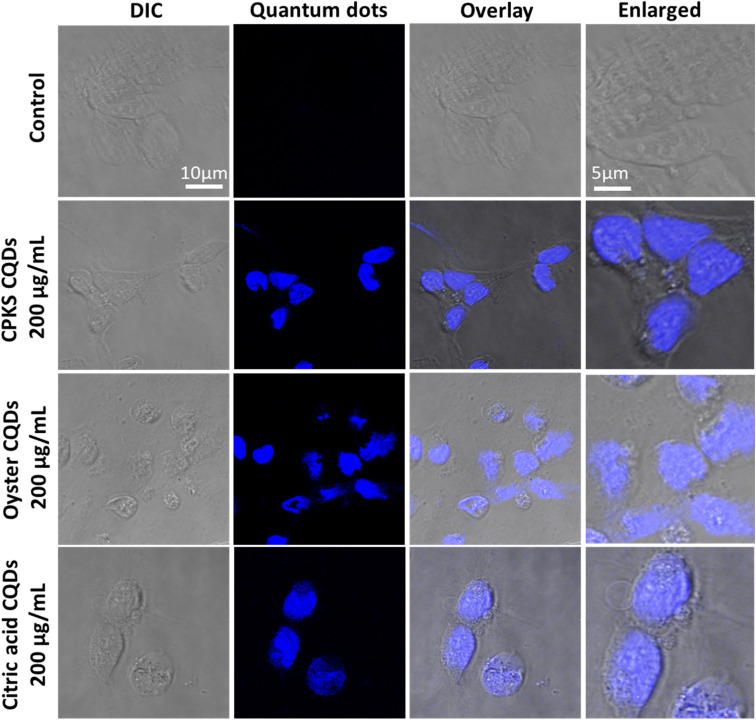
*In vitro* imaging studies using CPKS, oyster, and citric acid-based CQDs with cardiomyocyte cells.

In the case of cardiomyocytes cells (Fig. 15), CPKS-based CQDs do not show any cytotoxicity up to 600 μg mL^−1^ until 48 h; 800 μg mL^−1^ and 1000 μg mL^−1^ show 25% toxicity after 48 h. Oyster-shell based CQDs show no cytotoxicity up to 800 μg mL^−1^ after 24 hours, but 10% of cell death happened when we used 1000 μg mL^−1^. But 48 h later, oyster shell-based CQDs showed 50% toxicity at a higher 1000 μg mL^−1^ concentration. No toxicity was observed for CA-based CQDs up to 600 μg mL^−1^. But 1000 μg mL^−1^ shows 35% of cell toxicity was observed. Fig. 14 shows the cellular uptake of CQDs by cardiomyocyte cells. The cardiomyocytes cells show very good cellular uptake throughout the cardiomyocytes cells. We used 200 μg mL^−1^ for CPKS and oyster shell-based CQDs but only 100 μg mL^−1^ for citric acid CQDs due to high-level fluorescence capacity. We observed after 24 h of incubation with CQDs, even though we could not see cell morphology changes. These results convinced cell viability studies with 200 μg mL^−1^ of CQDs were nontoxic to cardiomyocyte cells.

### 3.7 CDs uptake in an *in vivo* model (medaka egg/larvae toxicity study)

**Fig. 16:**
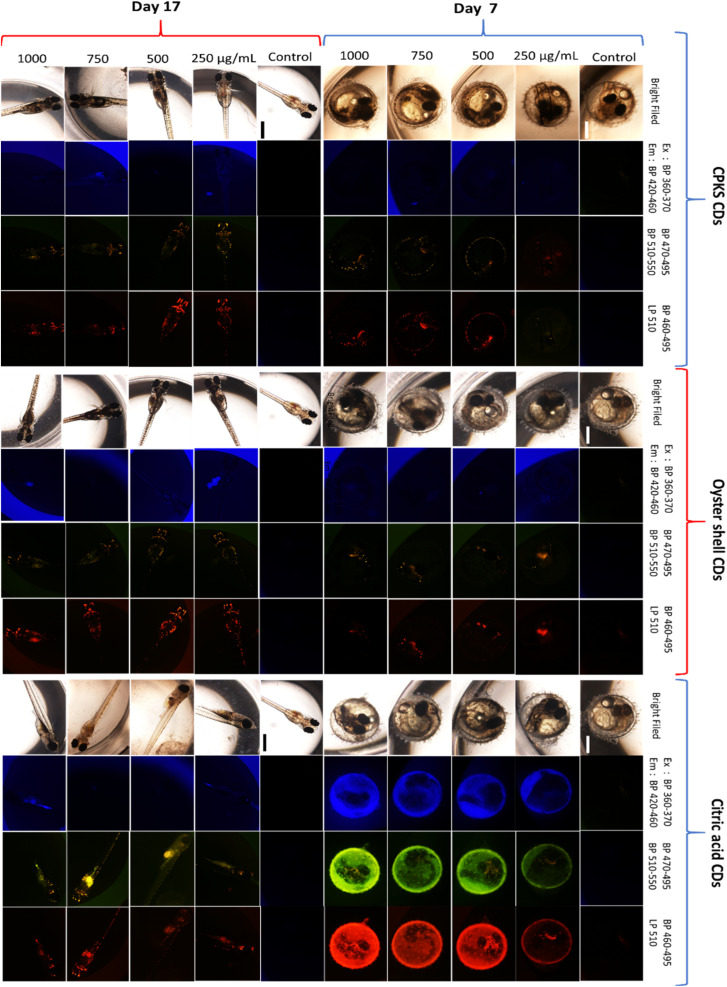
Medaka egg (day 7) and medaka fish (day 17) with increasing concentrations of QDs 250, 500, 750, and 1000 μg mL^−1^. Three eggs were used for each QDs concentration (*n* = 3).

To verify the *in vivo* staining capability of CQDs, we assessed their uptake in the Japanese medaka fish model during the embryo and larvae stages. We collected the eggs from the fish tank and then transferred them to 96-well plates. After that, we incubated them with 200 mL of sterile and nuclease-free water. After incubating the medaka eggs for 24 h, we introduced CQDs at 250, 500, 750, and 1000 μg mL^−1^ concentrations. We then utilized confocal microscopy to investigate whether the eggs could absorb the CQDs. By Fig. 16, CPKS, oyster shell, and CA-based CQDs are present within the medaka fish egg (day 7) without using vectors or transfection agents. We used three different channels to observe the fluorescence of CQDs, and all the medaka eggs looked healthy. We can see the CQD's color from yellow and red channels compared with blue channels. The results clearly demonstrate that even at concentrations as high as 1000 μg mL^−1^, the fish embryo treated with CQDs exhibited no abnormalities. Furthermore, by day 17, the larvae showed normal development and excellent fluorescence distribution, which indicates the exceptional biocompatibility of CQDs for *in vivo* imaging purposes. In between egg to larvae development, we observed medaka eggs/larvae development during days 9,11, and day 14 (Fig. S13–S21†). The research conducted by Kwok *et al.* has clearly shown that silver nanoparticles are mainly absorbed through the skin surface and gills of medaka larvae.^69^ Furthermore, Kashiwada *et al.* have discovered that most nano-sized particles in medaka eggs/larvae are absorbed through adsorption or accumulation.^70^ Therefore, it is highly probable that the uptake of CQDs by medaka eggs/larvae happens through adsorption/bioaccumulation or the gills. It is important to note that CQDs differ from silver nanoparticles in that they lack toxic properties, making them safe for both living organisms and cells.^71^

## 4. Conclusion

We created three types of CQDs by utilizing biomass waste materials like palm shells, oyster shells, and CA. Our team then proceeded to conduct a bioimaging experiment to determine cellular uptake. When comparing citric acid to the other two CQDs, it was found that the latter has more excellent stability under 365 nm ultraviolet light. Additionally, we optimized the fluorescence emission of each material by adjusting the EDA concentrations. We have thoroughly evaluated and chosen the most effective concentrations for every CQDs, guaranteeing that they remain under 10 nm in size. We conducted cell viability assays on all the CQDs and observed no cytotoxicity until 24 h of incubation. However, after 48 h, we noticed cell toxicity, specifically with iPS cells when exposed to citric acid, showing 80% cell toxicity. Compared to citric acid CQDs, the toxicity of biomass-derived CQDs is minimal in the first 24 h. After 48 h, it was noticed that oyster CQDs showed cytotoxicity at higher concentrations when tested with HeLa cells and cardiomyocytes. Based on a cell imaging experiment, it was observed that CQDs can enter the mitochondria of HeLa cells while also being taken up by iPS cells and cardiomyocytes without causing any changes in their morphology. An *in vivo* experiment on Japanese medaka fish confirmed that CQDs remain biocompatible even after a 17 day incubation period. The larvae have successfully healthily hatched from their eggs. There is hope that CQDs produced from biomass waste will have practical applications for diagnosis and treatment in the future.

## Author contributions

N. A. research planning and management, project supervision, synthesis of CQDs, characterization, analysis and draft manuscript, S. C. research planning and management, project supervision, application, analysis and draft manuscript, M. K. application and analysis, F. E. application and analysis, N. M. B. synthesis of CQDs, characterization, analysis, Z. A. Z. characterization and analysis, S. N. M. S. characterization and analysis, R. H. S. project supervision, S. K. and V. T. review and editing, J. A. project supervision, analysis and manuscript review and G. N. P. project supervision and manuscript review and editing.

## Conflicts of interest

The authors declare no competing interests.

## Supplementary Material

RA-013-D3RA05840A-s001
