# Supplementary material for: Development of biomass waste-based carbon quantum dots and their potential application as non-toxic bioimaging agents
Source: RSC Adv. 2023 Sep 25;13(40):28230–49. doi: 10.1039/d3ra05840a (PMC10518661; doi:10.1039/d3ra05840a)
Supplement: RA-013-D3RA05840A-s001 [file RA-013-D3RA05840A-s001.pdf]

Electronic Supplementary Material (ESI) for RSC Advances.
This journal is © The Royal Society of Chemistry 2023

Supporting Information

Development of biomass waste-based carbon quantum dots and their potential application as non-toxic bioimaging agents

Norhidayah Abu[1,2*], Shanmugavel Chinnathambi[3*], Mahima Kumar[3], Fatemeh Etezadi[3], Noremylia Mohd Bakhori[2], Zuhana Ahmad Zubir[2], Shahrul Nizam Md Salleh[2], Rafidah Hanim Shueb[1], Subramani Karthikeyan[4], Vaijayanthi Thangavel[3], Jaafar Abdullah[5*] and Ganesh N. Pandian[3*,]

[1]Department of Medical Microbiology & Parasitology, School of Medical Sciences, Universiti Sains Malaysia, Health Campus, Kubang Kerian 16150, Kelantan, Malaysia

[2]Advanced Materials Research Centre (AMREC), SIRIM Berhad, Lot 34, Jalan Hi-Tech 2/3, Kulim, Hi-Tech Park, 09000 Kulim, Malaysia

[3]Institute for Integrated Cell-Material Sciences, Institute for Advanced Study, Kyoto University, Kyoto, Japan

[4]Centre for Healthcare Advancement, Innovation and Research, Vellore Institute of Technology, Chennai, 600 127, India

[5]Department of Chemistry, Faculty of Science, Universiti Putra Malaysia, UPM Serdang, Serdang 43400, Selangor, Malaysia

C.A. E-mail: nhidayah@sirim.my; chinnathambi.shanmugavel.8s@kyoto-u.ac.jp; jafar@upm.edu.my; namasivayam.ganeshpandian.5z@kyoto-u.ac.jp

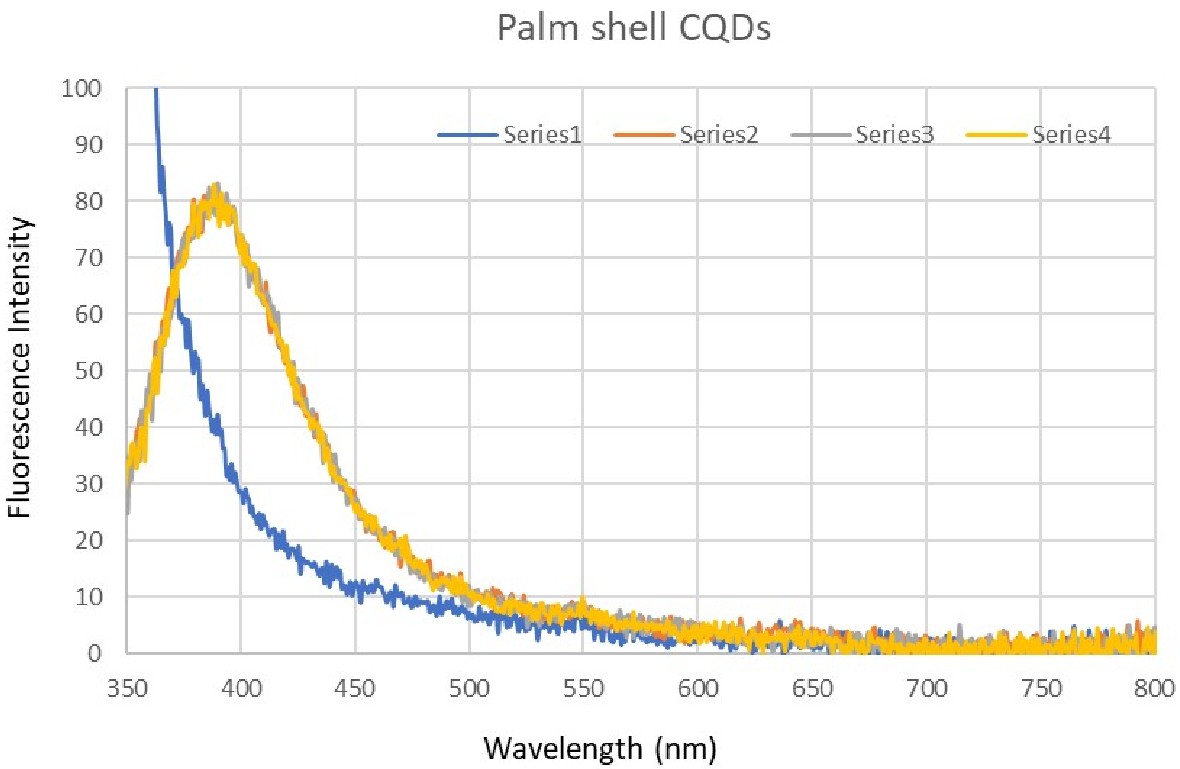

Sample (n=3)	**Quantum Yield (%)**	Abs	Peak Wavelength	Peak Count	Peak FWHM
Reference (Series 1)	**------**	-----	371.88	65.27	141.66
Palm shell CDs 1 (Series 2)	**2.4**	0.914	385.5	81.26	74.74
Palm shell CDs 2 (Series 3)	**2.4**	0.914	390.04	83.2	69.04
Palm shell CDs 3 (Series 4)	**2.3**	0.914	388.53	82.88	71.34

Fig.S1 Palm shell carbon quantum dots and their absolute quantum yield measurements: Figure and its related parameters.

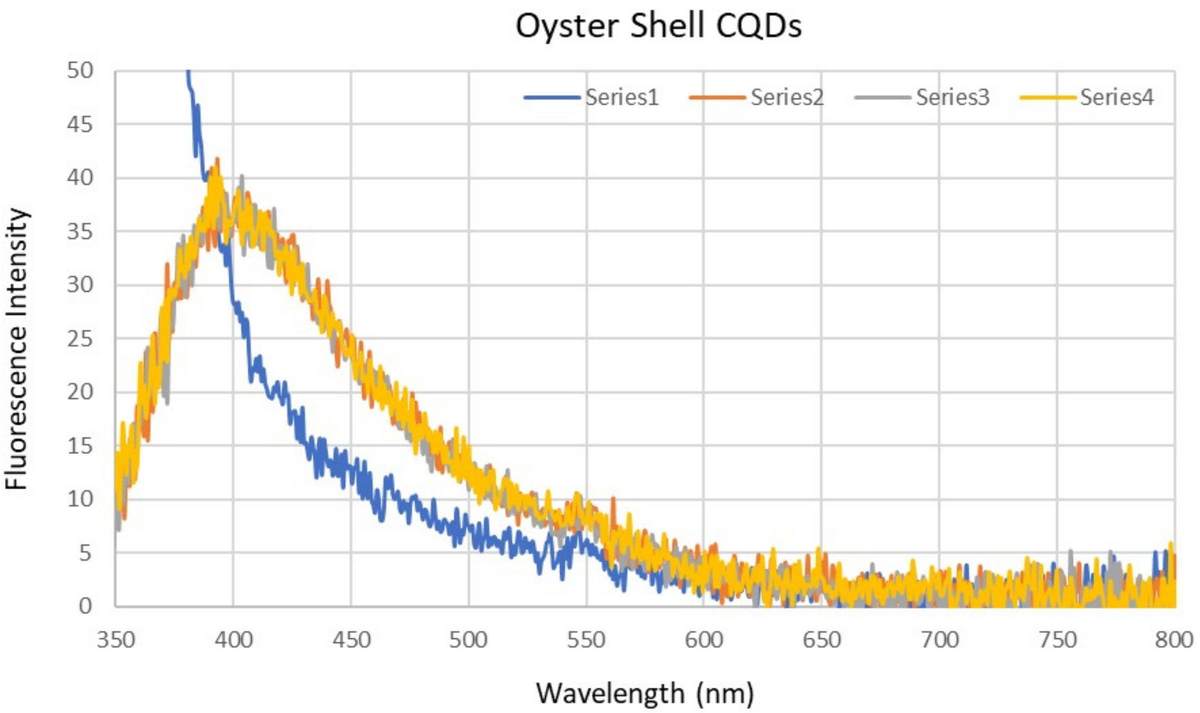

Sample (n=3)	**Quantum Yield (%)**	Abs	Peak Wavelength	Peak Count	Peak FWHM
Reference (Series 1)	**---**	---	392.31	36.51	423.69
Oyster shell CDs 1 (Series 2)	**1.4**	0.967	393.07	41.72	89.03
Oyster shell CDs 2 (Series 3)	**1.3**	0.967	403.65	40.09	90.84
Oyster shell CDs 3 (Series 4)	**1.4**	0.967	392.31	41.03	88.79

Fig. S2 Oyster shell carbon quantum dots and their absolute quantum yield measurements: Figure and its related parameters.

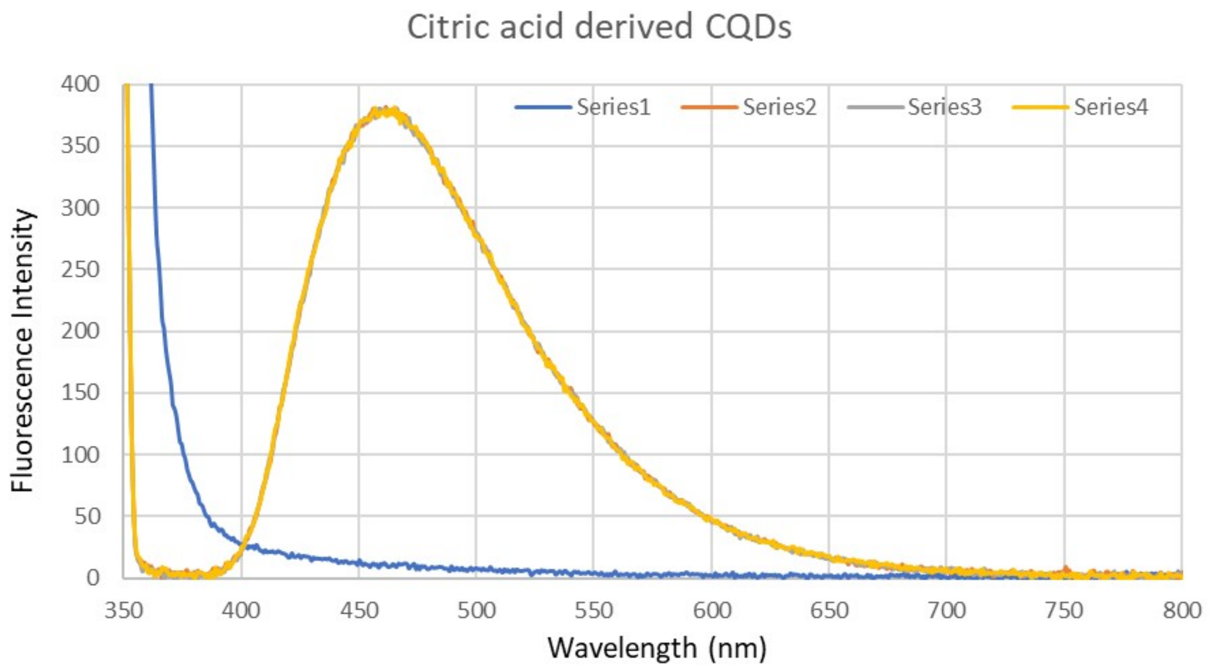

Sample (n=3)	**Quantum Yield (%)**	Abs	Peak Wavelength	Peak Count	Peak FWHM
Reference (Series 1)	**---**	---	406.67	26.74	165.51
CA CDs 1 (Series 2)	**22.1**	0.986	461.72	381.47	102.29
CA CDs 2 (Series 3)	**22.1**	0.986	466.24	381.01	102.33
CA CDs 3 (Series 4)	**22.1**	0.986	465.49	381.46	103.04

Fig. S3 Citric acid carbon quantum dots and their absolute quantum yield measurements: Figure and its related parameters.

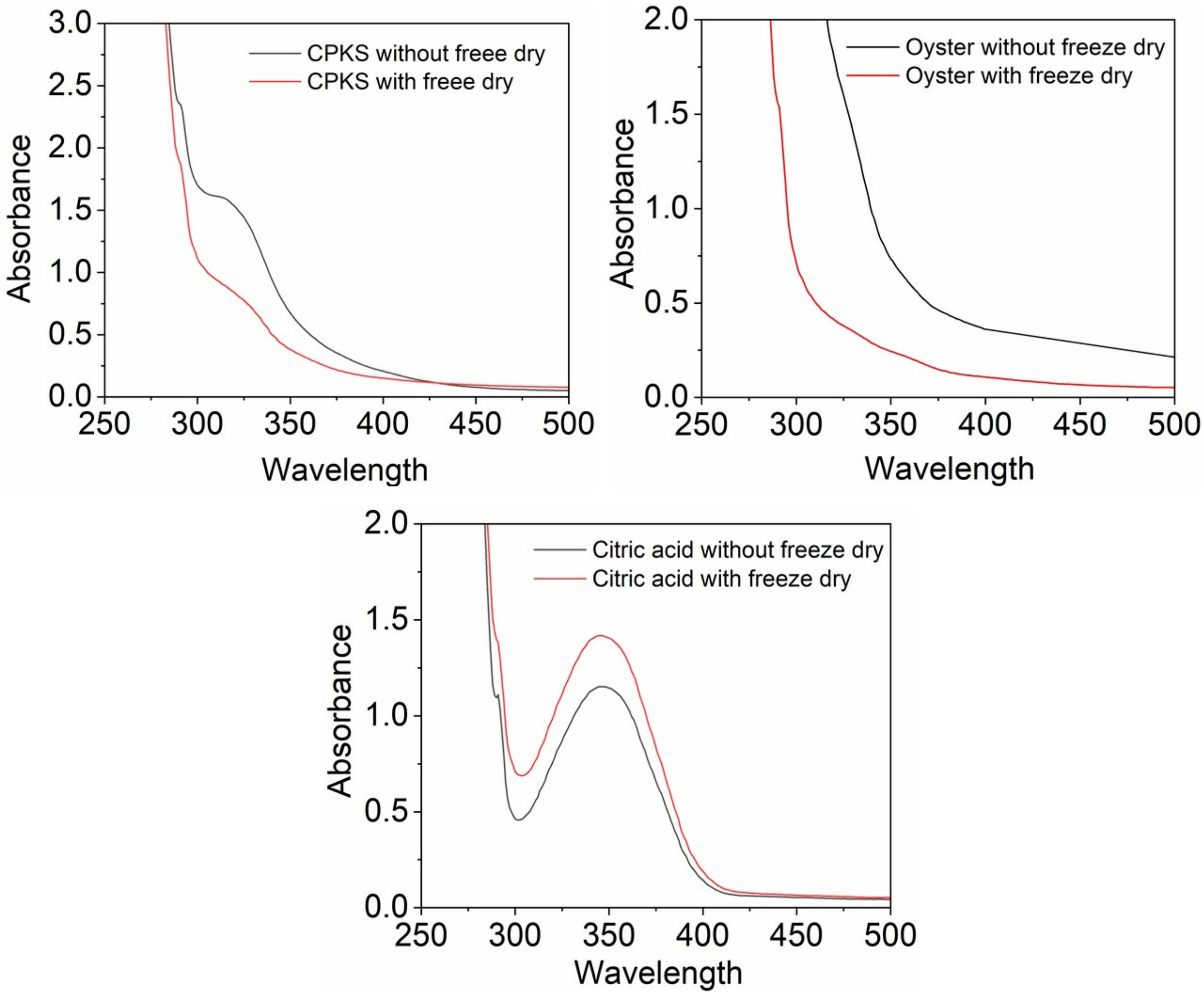

Fig. S4 Effect of freeze-dry process vs. absorbance of carbon dots

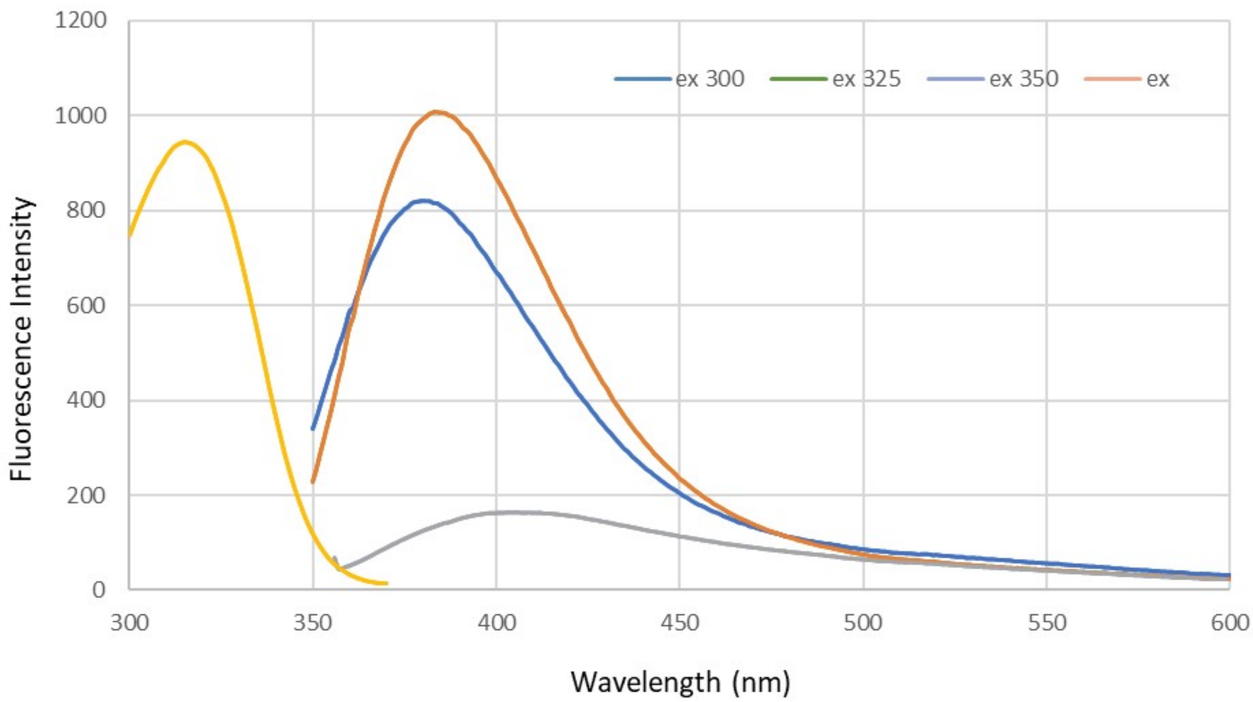

Fig. S5 The excitation spectrum of CPKS carbon quantum dots (yellow line) and its emission spectra with various wavelengths (excitation at 300 nm, 315nm, 325nm)

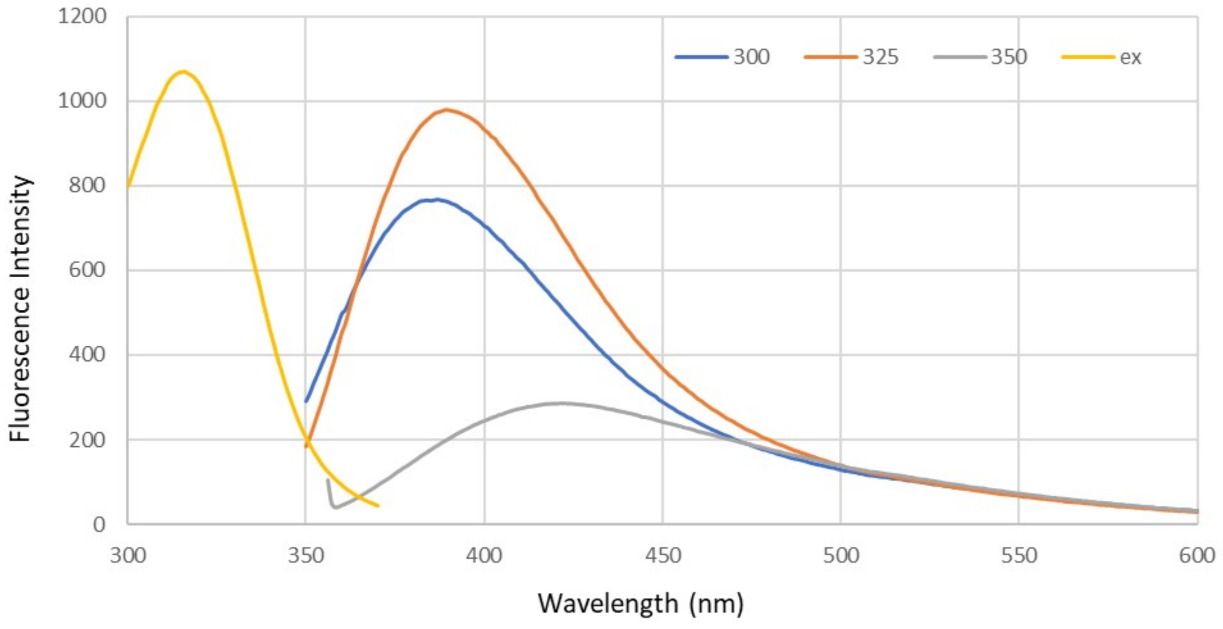

Fig.S6 The excitation spectrum of oyster carbon quantum dots (yellow line) and its emission spectra with various wavelengths (excitation at 300 nm, 325nm, 350nm)

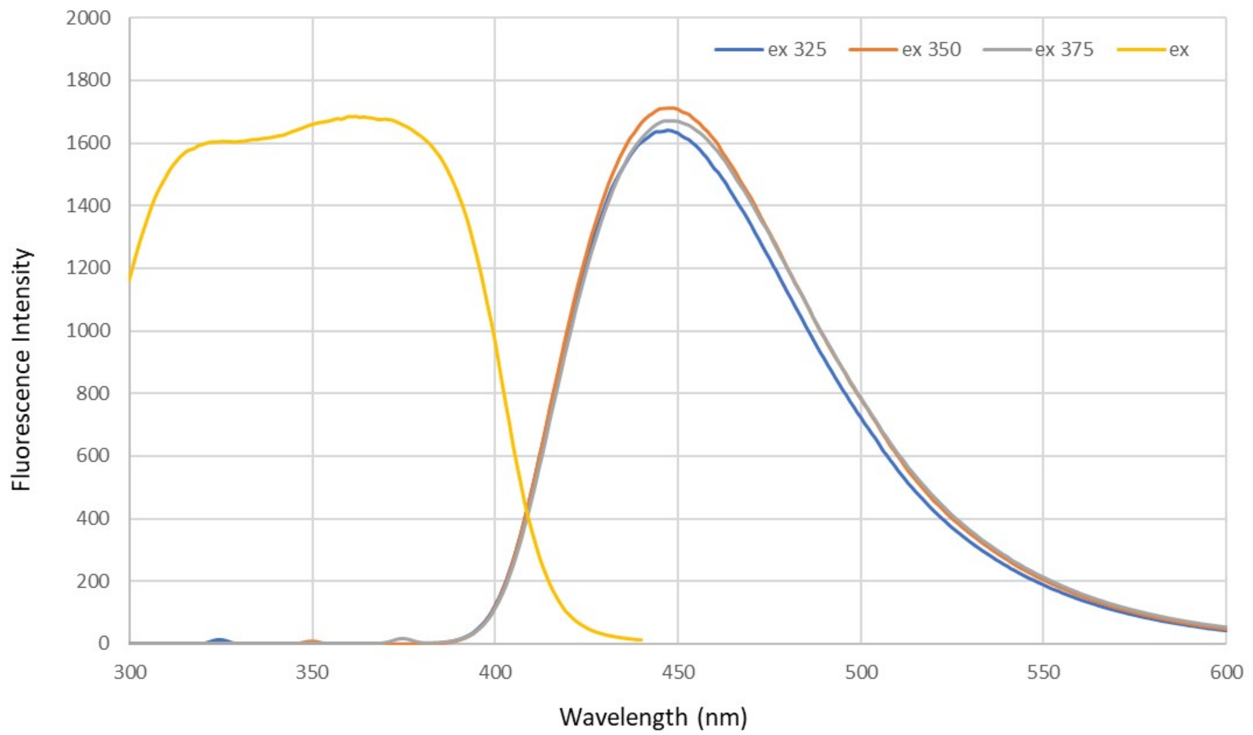

Fig. S7 The excitation spectrum of citric acid carbon quantum dots (yellow line) and its emission spectra with various wavelengths (excitation at 325 nm, 350nm, 375nm)

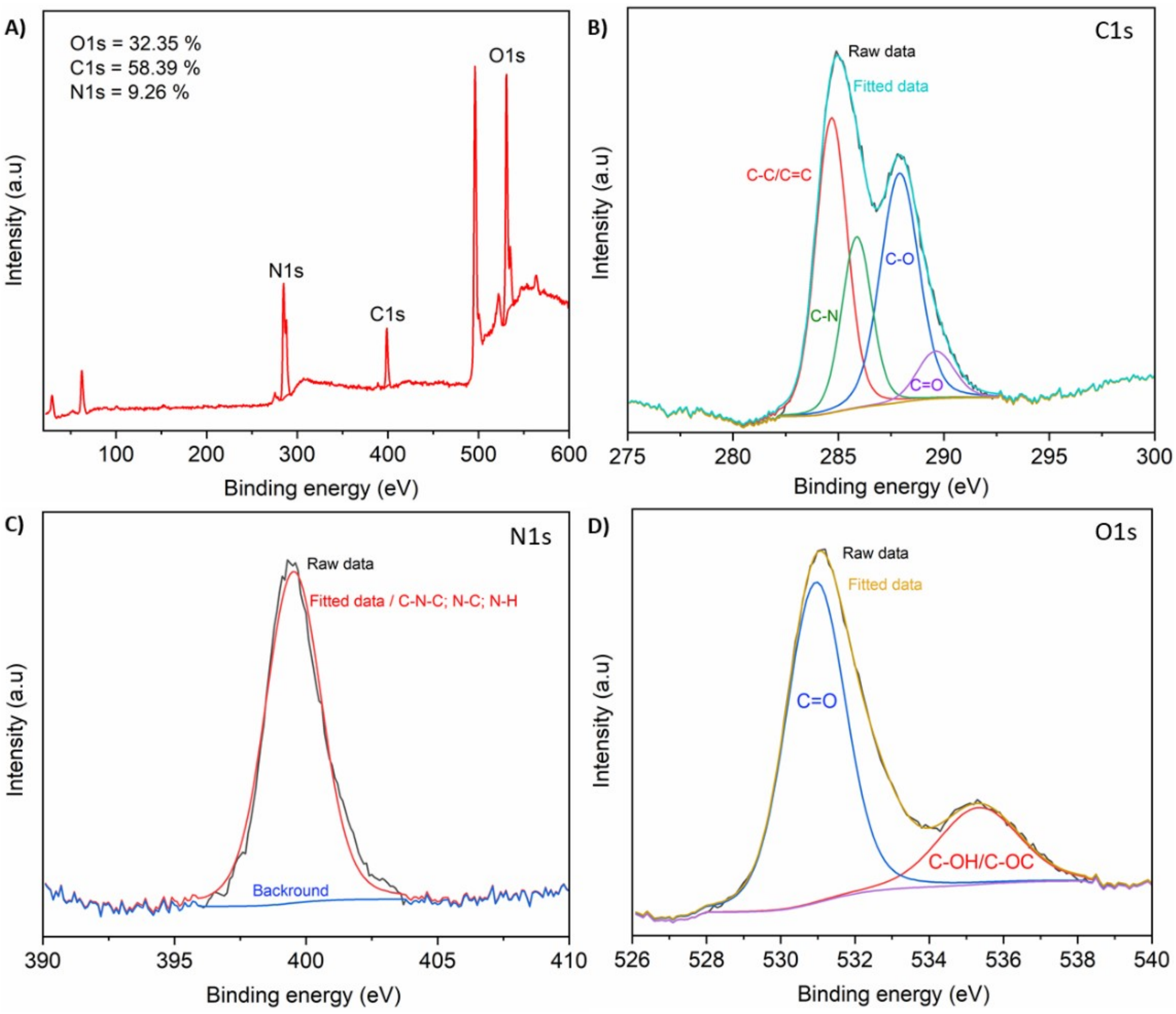

Fig. S8 XPS full-survey spectrum of CQDs from CPKS (a); high-resolution XPS of the C1s (b), N1s (c), and O1s (d) spectra.

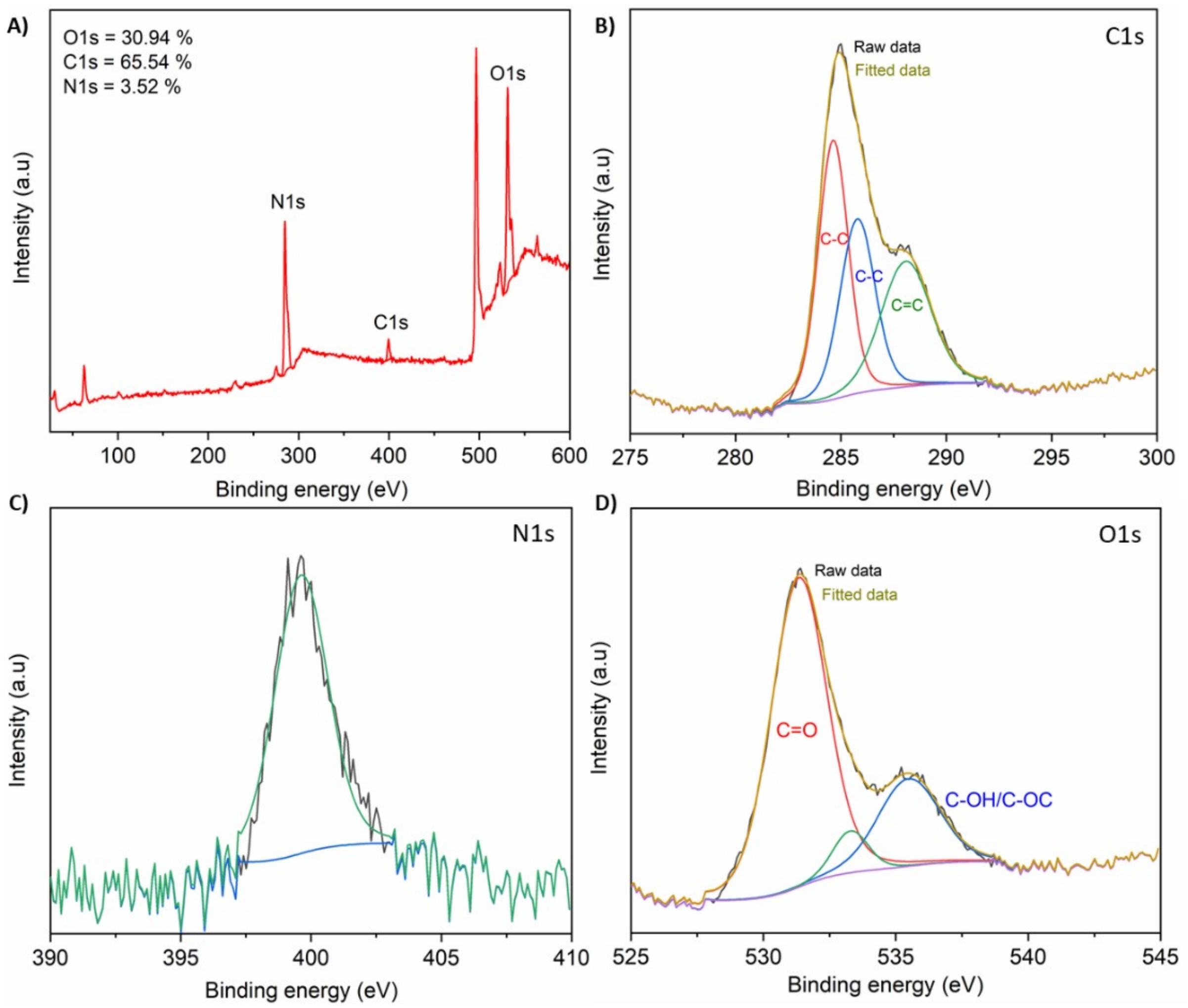

Fig. S9 XPS full-survey spectrum of CQDs from Oyster shell (a); high-resolution XPS of the C1s (b), N1s (c), and O1s (d) spectra.

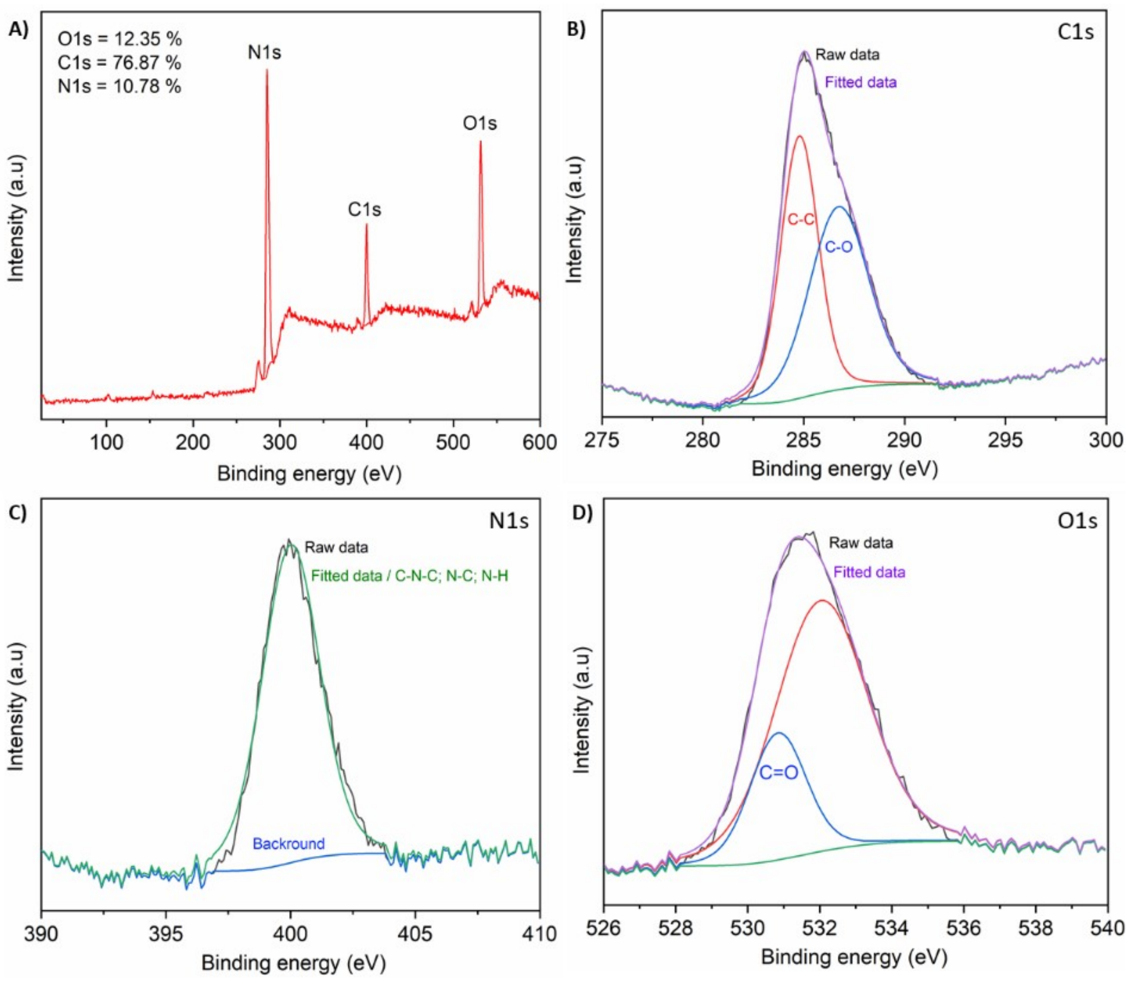

Fig. S10 XPS full-survey spectrum of CQDs from citric acid (a); high-resolution XPS of the C1s (b), N1s (c), and O1s (d) spectra.

Table.S1 C1s, N1s, and O1s and the relative atomic percentages of these peaks

Type of CQDs	Binding energy (eV)							Element content (%)		
	C-C, C-H	C-N	C-N, C-O	C=O, OH-C-H	O=C	O-C	R-NH-R	C	O	N
CPKS	284.68	285.87	287.90	289.61	530.95	532.35	399.3	58.39	32.35	9.26
Oyster shell	284.63	285.78	288.08		531.34	533.29	399.11	65.54	30.94	3.52
Citric acid	284.79	286.73			530.84	532.03	399.92	76.87	12.35	10.78

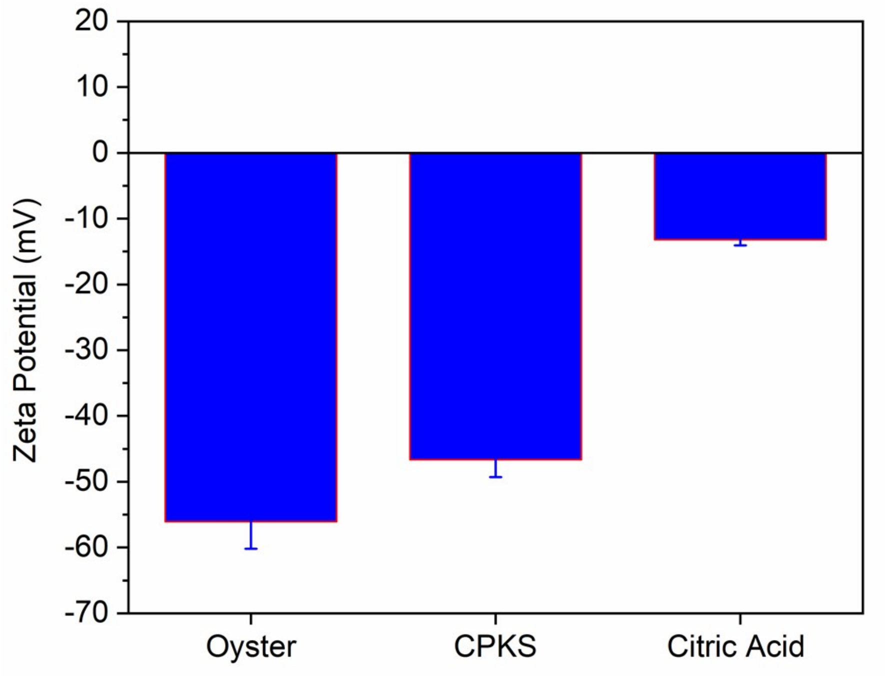

Fig. S11 Zeta potential measurement of synthesized QDs from three different sources.

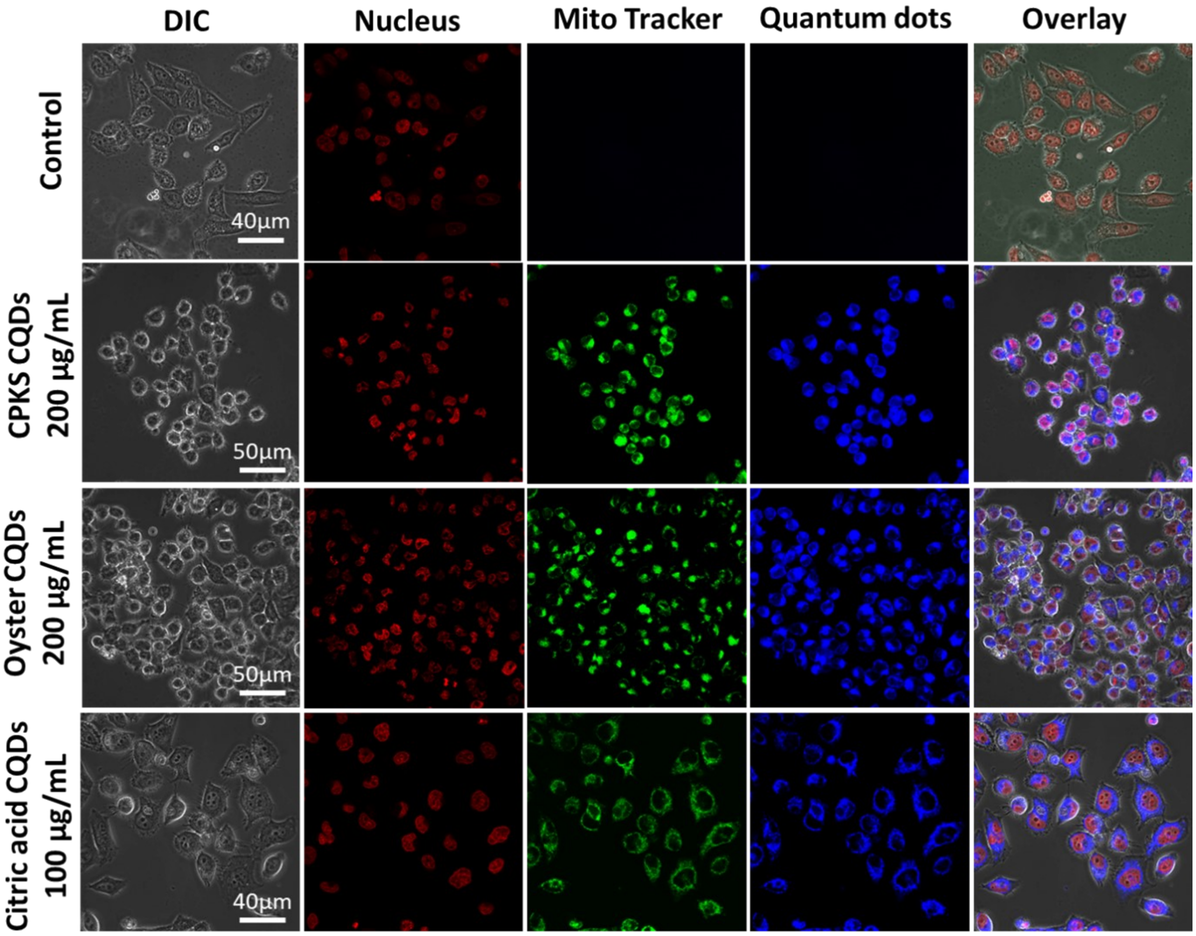

Fig. S12 Mitochondrial localization using CPKS, Oyster, and Citric acid-based CQDs with HeLa cells.

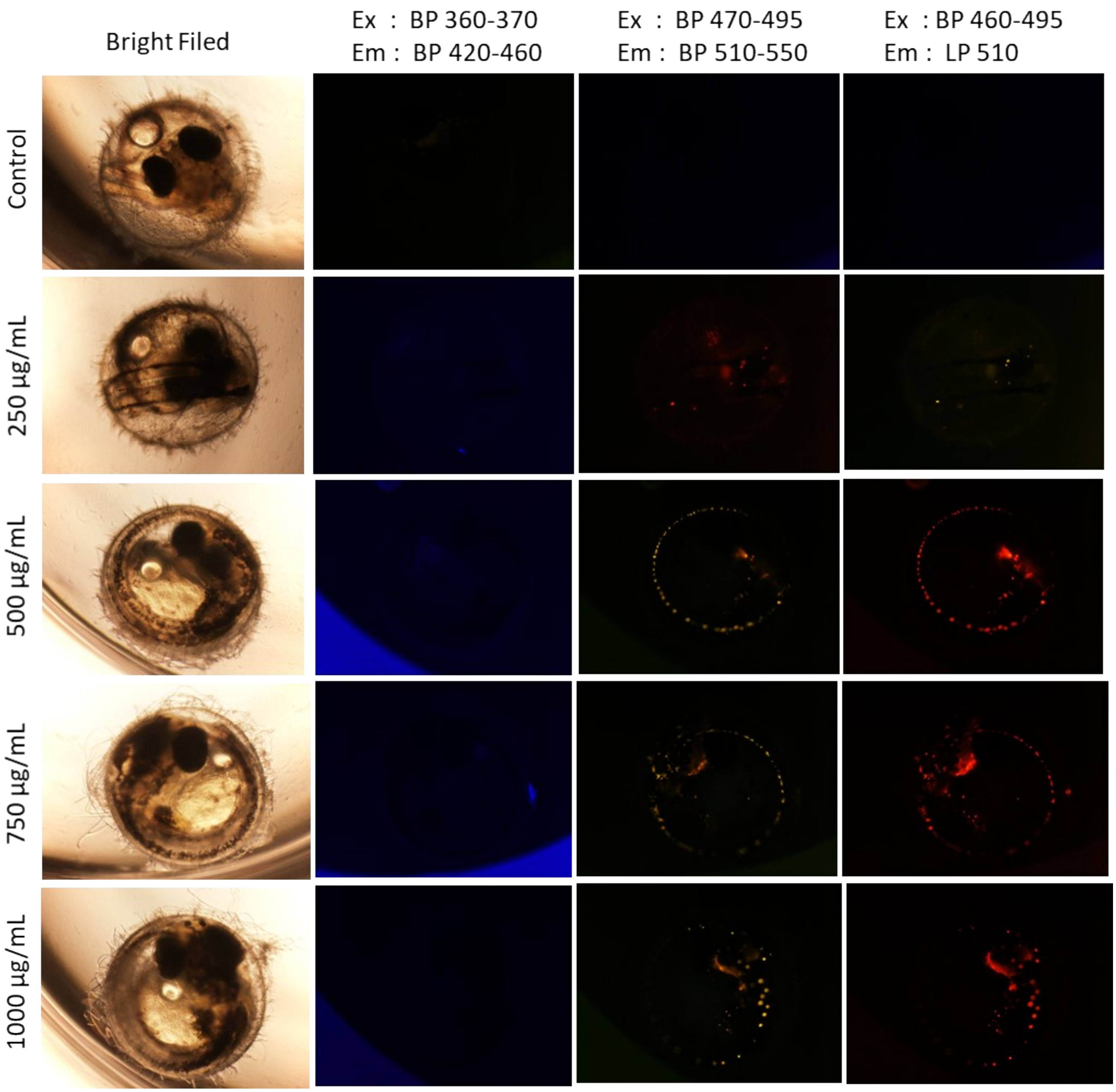

Fig. S13 Medaka egg (day-9) with increasing concentrations of **CPKS - QDs** 250, 500, 750 and 1000 μg mL^{-1}. Three eggs were used for each micelle concentration ± S.E. ($n = 3$).

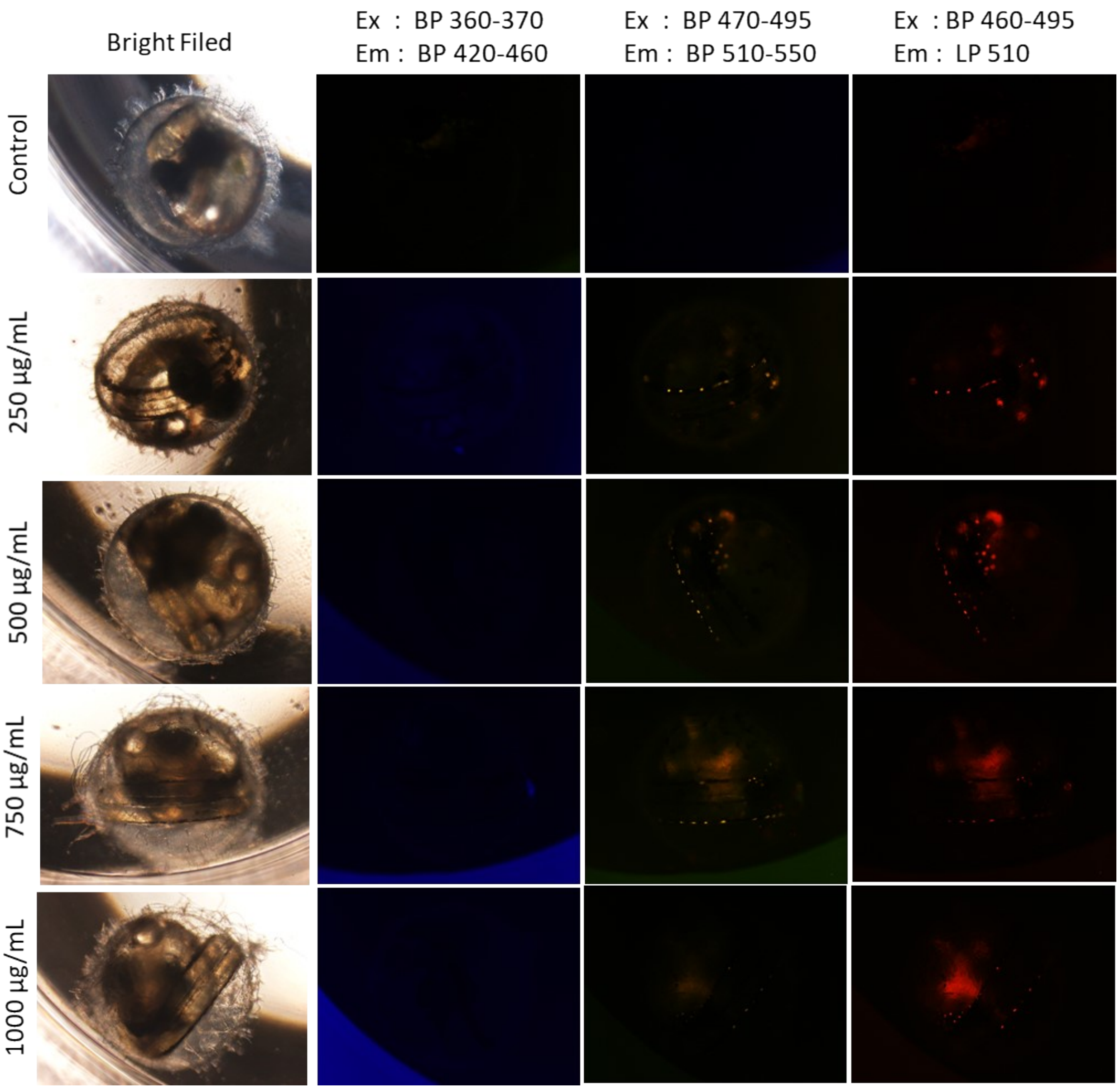

Fig. S14 Medaka egg (day-11) with increasing concentrations of **CPKS - QDs** 250, 500, 750 and 1000 μg mL^{-1}. Three eggs were used for each micelle concentration ± S.E. (n = 3).

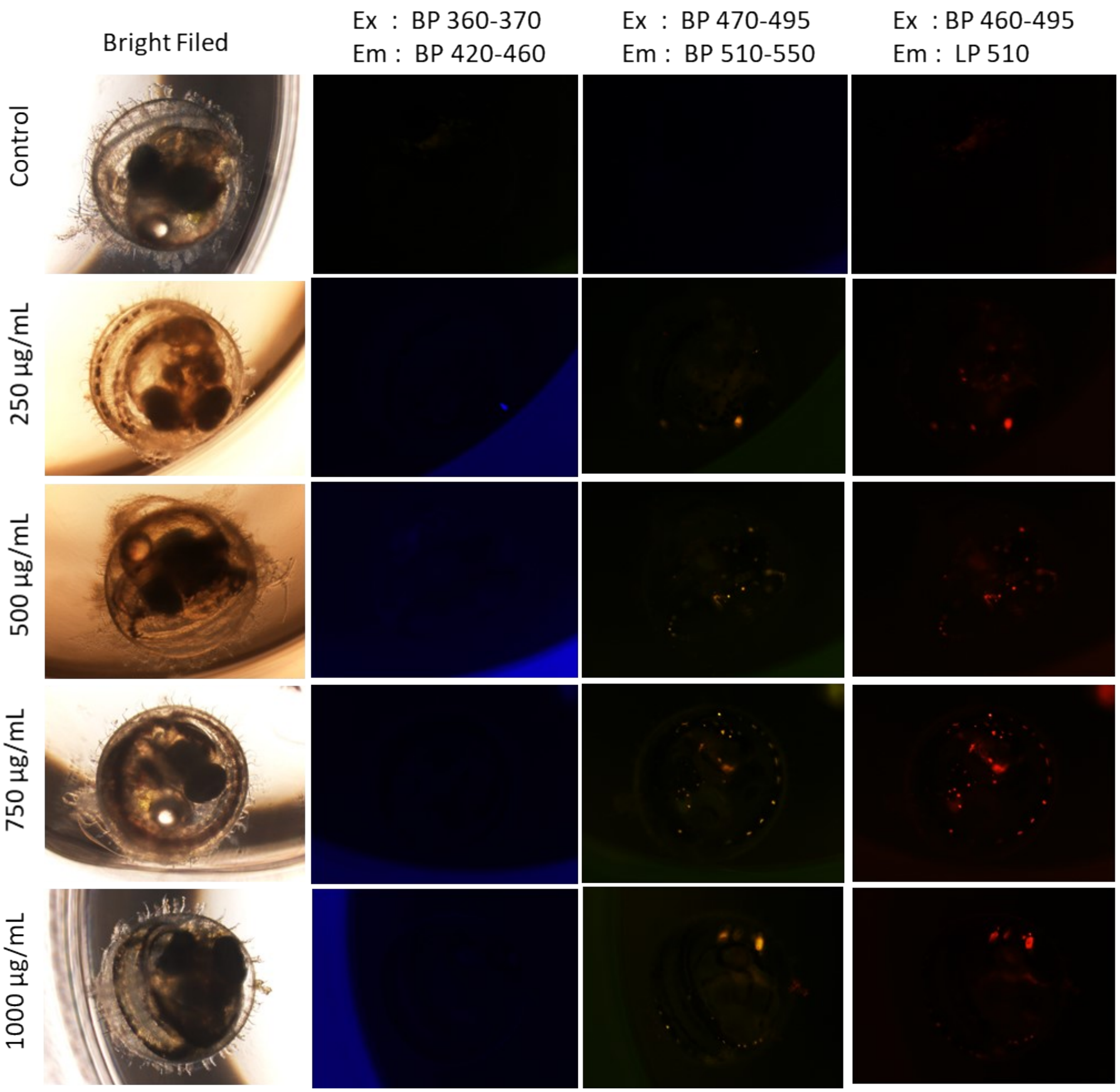

Fig. S15 Medaka egg (day-14) with increasing concentrations of **CPKS - QDs** 250, 500, 750 and 1000 μg mL^{-1}. Three eggs were used for each micelle concentration ± S.E. (n = 3).

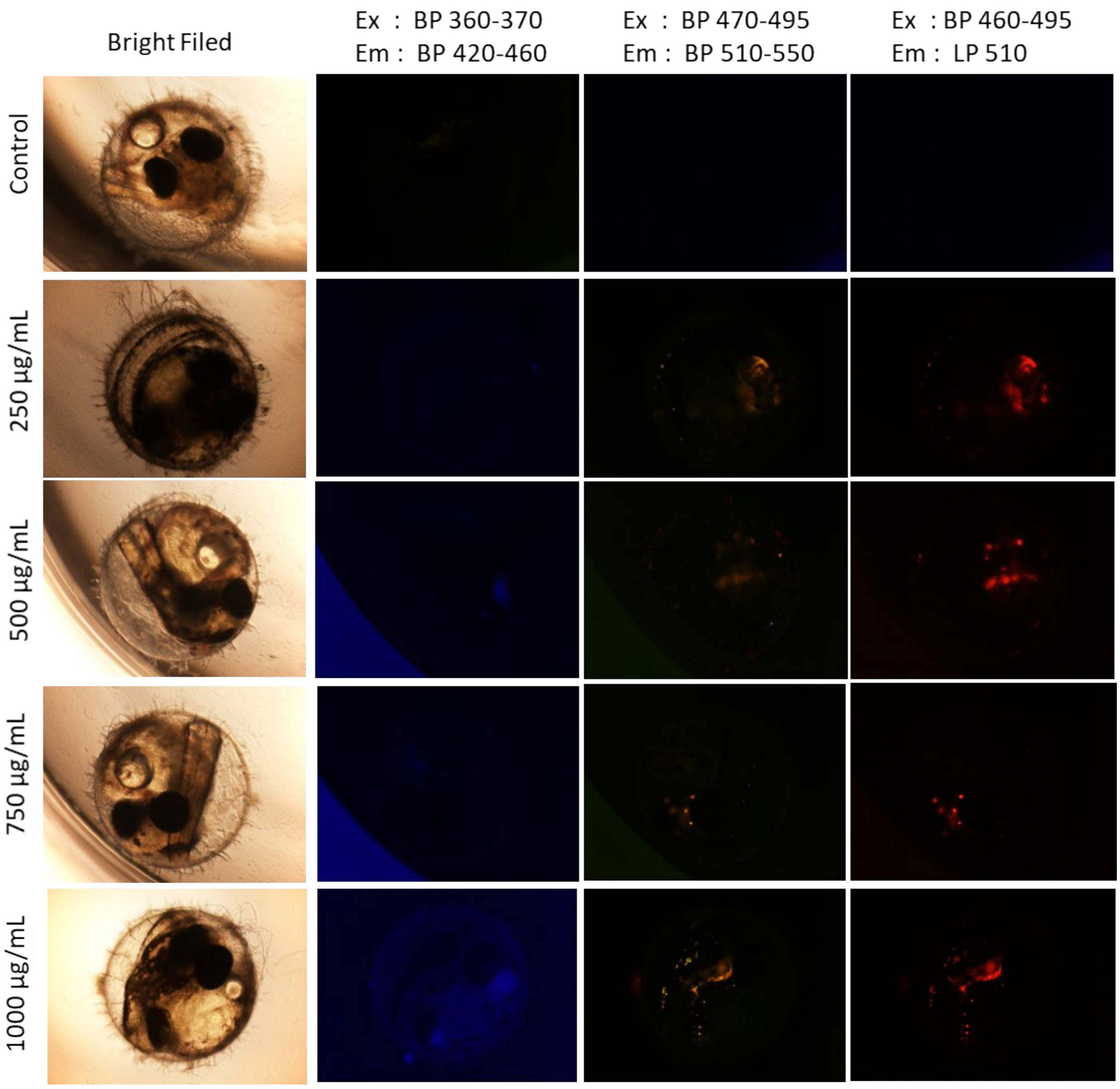

Fig. S16 Medaka egg (day-9) with increasing concentrations of **Oyster - QDs** 250, 500, 750 and 1000 μg mL^{-1}. Three eggs were used for each micelle concentration ± S.E. (n = 3).

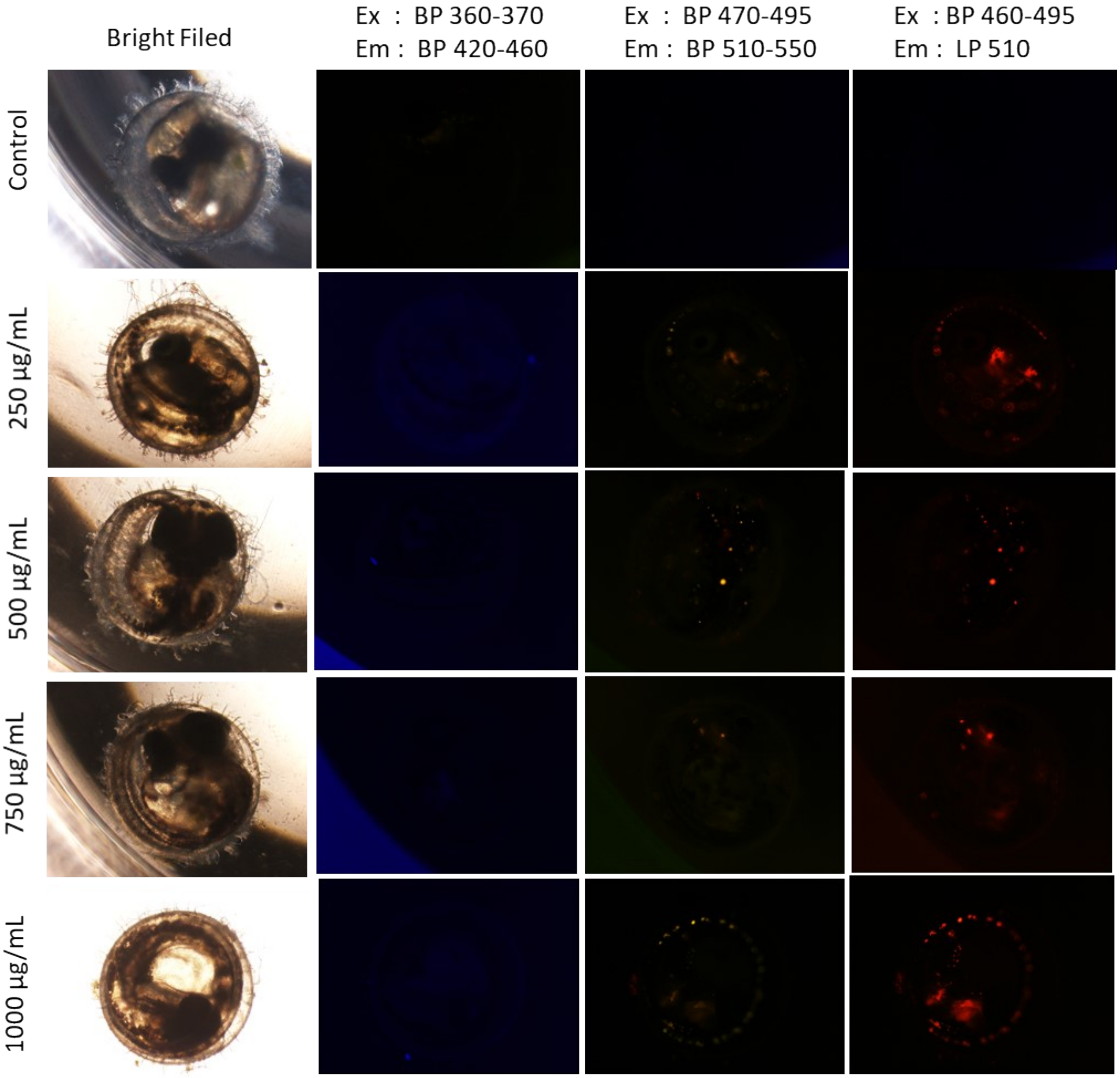

Fig. S17 Medaka egg (day-11) with increasing concentrations of **Oyster - QDs** 250, 500, 750, and 1000 µg mL^{-1}. Three eggs were used for each micelle concentration ± S.E. (n = 3).

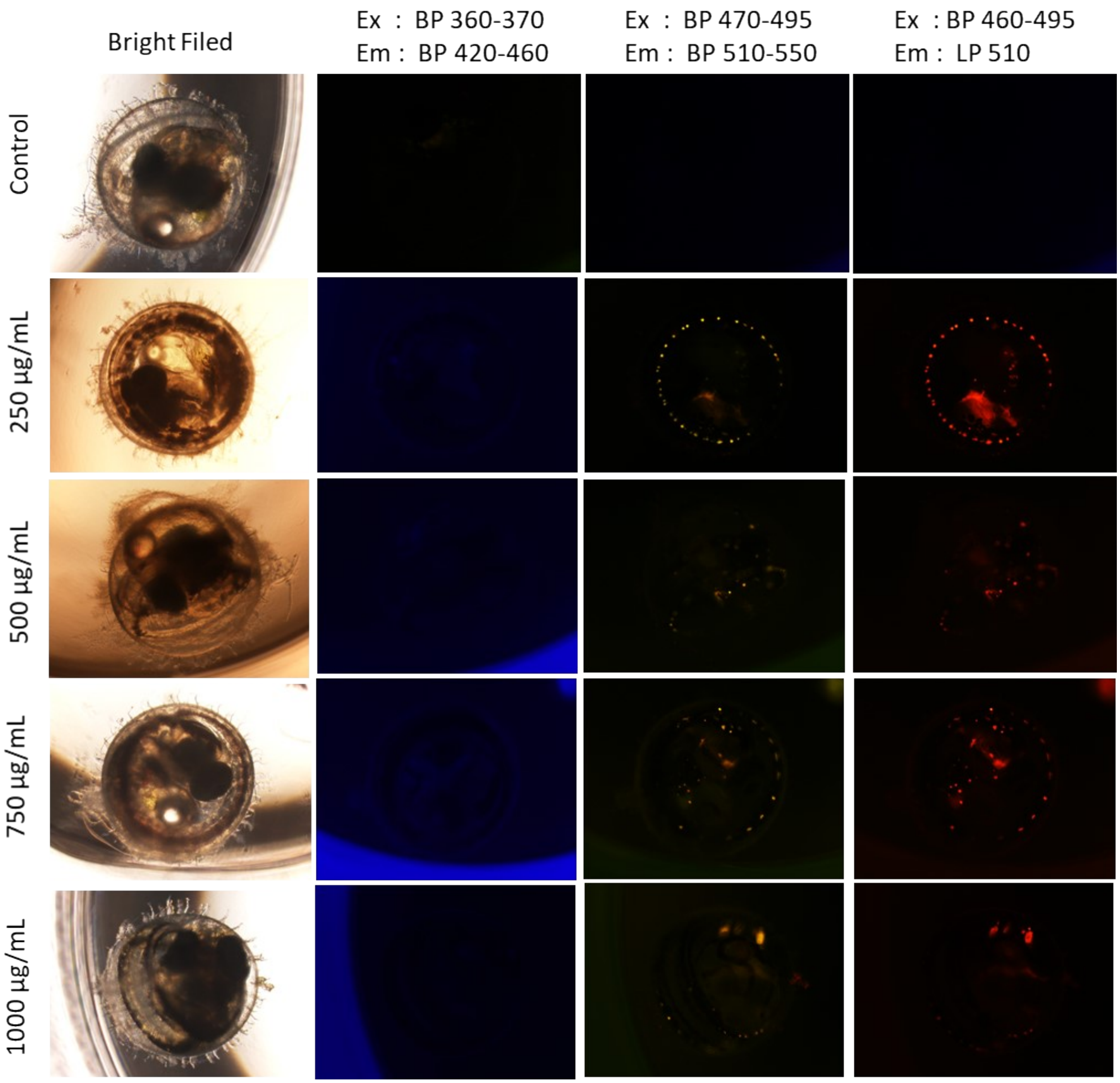

Fig. S18 Medaka egg (day-14) with increasing concentrations of **Oyster - QDs** 250, 500, 750 and 1000 µg mL^{-1}. Three eggs were used for each micelle concentration ± S.E. (n = 3).

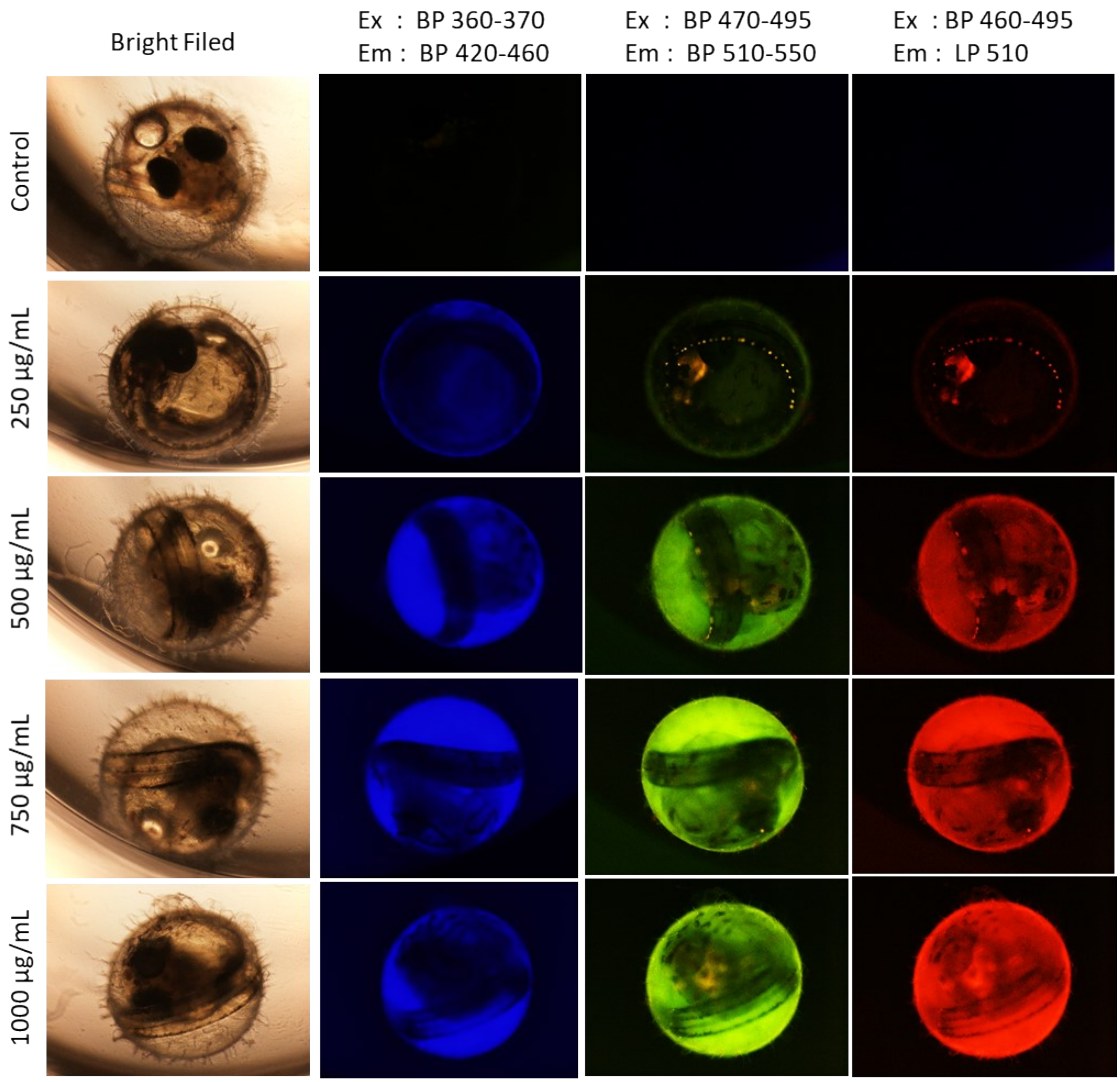

Fig. S19 Medaka egg (day-9) with increasing concentrations of **CA-QDs** 250, 500, 750 and 1000 μg mL^{-1}. Three eggs were used for each micelle concentration ± S.E. (*n* = 3).

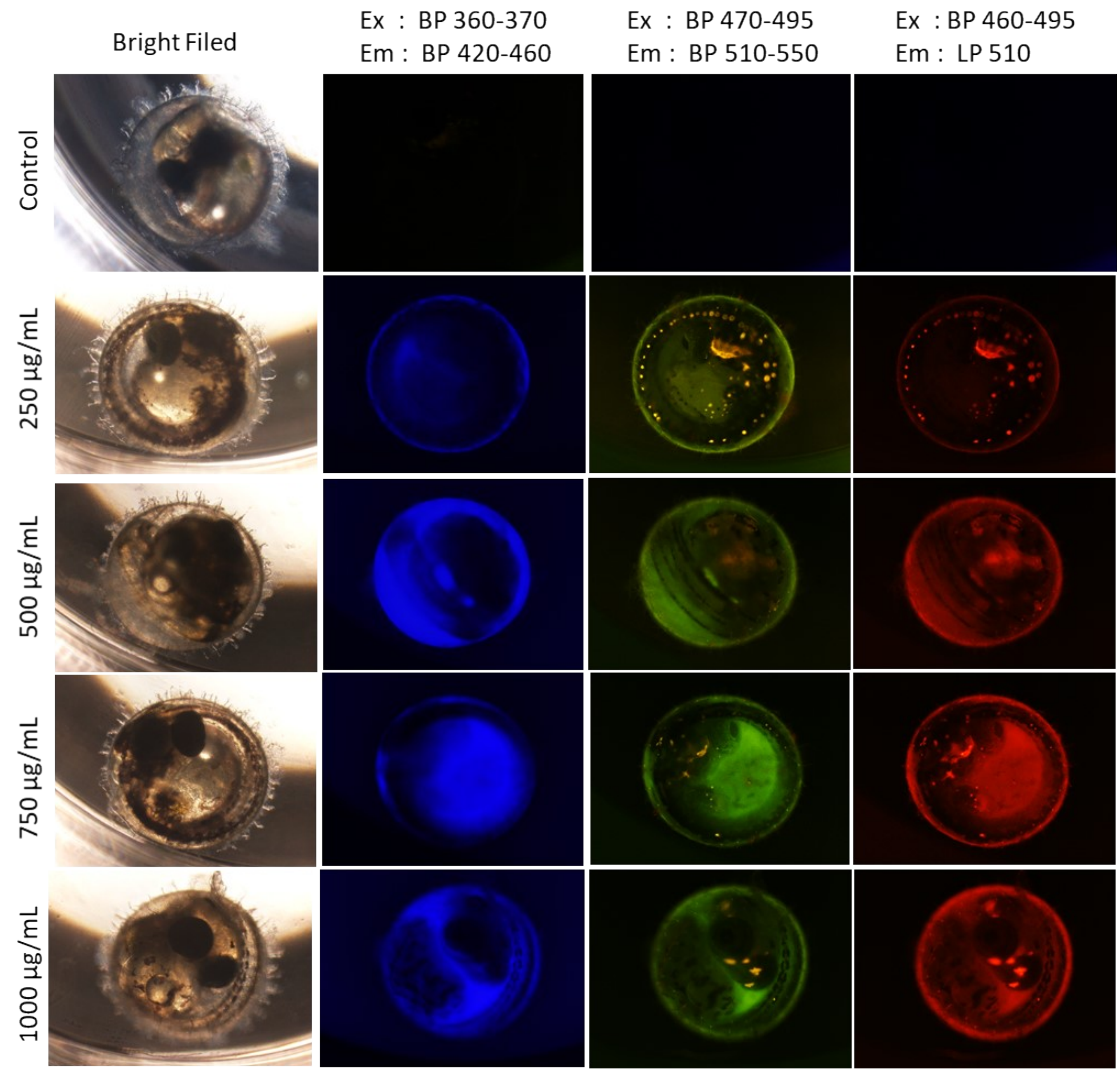

Fig. S20 Medaka egg (day-11) with increasing concentrations of **CA-QDs** 250, 500, 750, and 1000 μg mL^{-1}. Three eggs were used for each micelle concentration ± S.E. (*n* = 3).

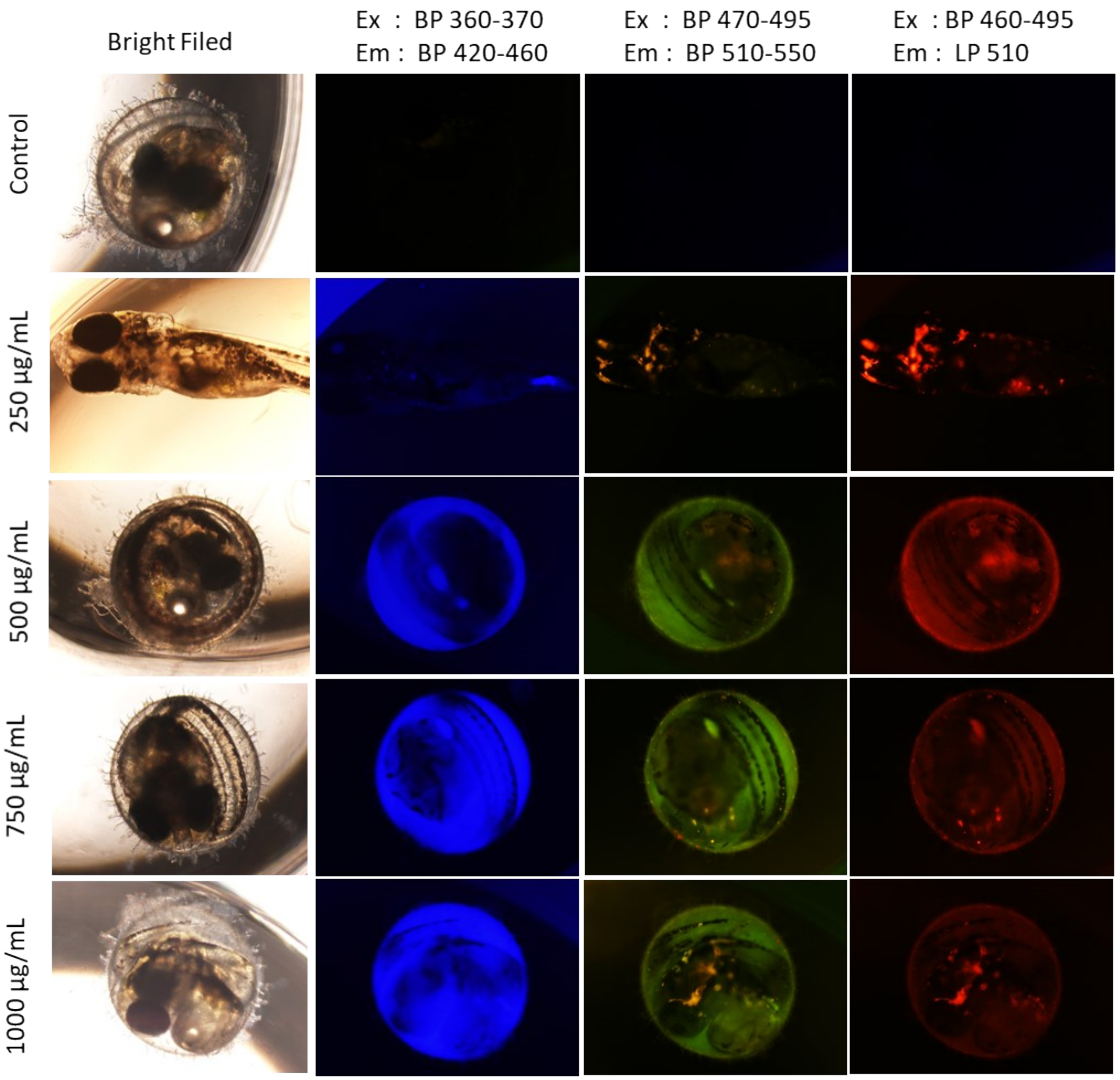

Fig. S21 Medaka egg (day-14) with increasing concentrations of **CA-QDs** 250, 500, 750 and 1000 µg mL^{-1}. Three eggs were used for each micelle concentration ± S.E. ($n = 3$).
